# Single‐Nucleus RNA Sequencing Identifies CREM as a Brake on Myofiber Hypertrophy in Chickens

**DOI:** 10.1002/advs.78534

**Published:** 2026-10-28

**Authors:** Shuang Gu, Xiyu Zhao, Zheyi Jiang, Chaoliang Wen, Chaoyi Wang, Qiulian Wang, Qiang Huang, Fangren Lan, Shenghui Wu, Junying Li, Congjiao Sun, Huadong Yin, Ning Yang

**Affiliations:** ^1^ National Engineering Laboratory for Animal Breeding Department of Animal Genetics and Breeding College of Animal Science and Technology China Agricultural University Beijing China; ^2^ State Key Laboratory of Swine and Poultry Breeding Industry College of Animal Science and Technology Sichuan Agricultural University Chengdu China; ^3^ Department of Animal Genetics and Breeding College of Animal Science and Technology Anhui Agricultural University Hefei China; ^4^ LC‐Bio Technology Co. Ltd. Hangzhou China

**Keywords:** chicken, myofiber hypertrophy, single‐nucleus RNA sequencing, skeletal muscle development, transcription factor

## Abstract

Myofiber hypertrophy is central to economically important agricultural traits and vertebrate development, yet the underlying cellular and molecular programs remain elusive. Chickens have undergone substantial divergence in muscle growth following decades of artificial selection, making them a unique model for dissecting these mechanisms at single‐cell resolution. Here, we performed singlenucleus RNA sequencing on 91 144 nuclei from postnatal chicken pectoralis major. Eight major conserved cell populations were resolved, with temporal dynamics exhibiting divergence between fast‐growing broilers and slow‐growing layers. A novel terminally differentiated myocyte subpopulation (*AK1* ^+^ myocytes) was markedly enriched in slow‐growing birds. Analysis revealed that accelerated myoblast expansion and delayed terminal differentiation are primary drivers of myofiber hypertrophy, supported by a pro‐growth niche involving IGF‐signaling FAPs and macrophage‐myogenic crosstalk. Further comprehensive analyses identified CREM as a master repressor of myofiber hypertrophy. Mechanistically, CREM directly binds and transcriptionally activates the *JUN* promoter, and both factors cooperatively sustain the expression of growth inhibitors MSTN, FBXO32, and TRIM63. In vivo and in vitro functional validation confirmed that CREM deletion promotes myofiber hypertrophy, increasing pectoralis major weight and percent by 29.96% and 20.50%, respectively. This study uncovers CREM as a novel brake on myofiber hypertrophy, offering fundamental insights into vertebrate skeletal muscle development.

## Graphical abstract

Single‐nucleus RNA sequencing on 91 144 nuclei from postnatal chicken pectoralis major between fast‐growing broilers (FG) and slow‐growing layers (SG) identifies the transcriptional factor CREM as a novel brake of myofiber hypertrophy. Mechanistically, functional validation experiments confirm that CREM activates the promoter of JUN and CREM deletion promotes myofiber hypertrophy, increasing pectoralis major weight and percent by 29.96% and 20.50%, respectively.

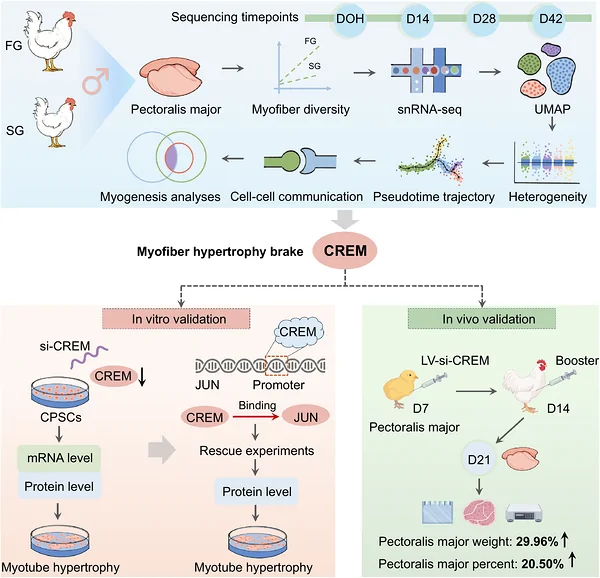

## 1 Introduction

Myofiber hypertrophy is a fundamental biological process underlying vertebrate skeletal muscle development, with broad relevance to metabolic homeostasis [1, 2], exercise physiology [3], and the pathogenesis of muscle atrophy [4]. In farm animals, myofiber hypertrophy is the primary driver of postnatal muscle mass accretion [5], directly determining growth performance and the capacity to provide high‐quality protein for human consumption. As an oviparous vertebrate, chicken has been an important model for developmental biology, due to the ease of accessing [6]. Notably, decades of intensive selection have generated an extraordinary divergence in muscle growth between fast‐growing broilers (FG) and slow‐growing layers (SG) [7], with the skeletal muscle growth rate of broilers approximately five times higher than that of layers [8]. This remarkable phenotypic contrast constitutes a uniquely powerful natural model for investigating the molecular mechanisms governing myofiber hypertrophy.

Studies in chickens have identified key regulators across multiple aspects of muscle development. *MiR‐16‐5p* and *SESN1* have been shown to govern myoblast proliferation, apoptosis, and differentiation [9]. Regarding myofiber hypertrophy, 3D genome maps of chicken skeletal muscle indicated that the alleles of *SMAD1* served as a positive regulator of myofiber size [10]. Myofiber type transformation has been associated with the transcriptional activity of TBP‐target genes and TBP protein [11]. Yet these bulk approaches cannot resolve the dynamic cellular heterogeneity, intercellular communication networks, or cell‐type‐specific transcriptional programs that constitute the muscle microenvironment of myofiber hypertrophy. Meanwhile, single‐nucleus RNA sequencing (snRNA‐seq) has emerged as a powerful approach to explore the cellular microenvironment at single‐cell resolution, substantially advancing our understanding of skeletal muscle biology in human disease contexts [12]. However, its application to the unique developmental dynamics of avian species remains limited. Recent single‐cell studies in poultry have focused on organogenesis in the heart [13], immune system [14], gonads and ovaries [15, 16], or localized muscle pathologies such as wooden breast [17]. Despite these advancements, a high‐resolution, time‐resolved single‐nucleus profiling capturing the transcriptional programs that drive postnatal myofiber hypertrophy in chickens remains to be established. As a critical component driving the rapid growth of broilers, the pectoralis major was selected as the target tissue, building upon our previous preliminary investigations at the bulk level [18]. Additionally, to capture the dynamic process of post‐hatching myofiber hypertrophy, we focused on four specific time points, day of hatch (DOH), day 14 (D14), D28, and D42, which represent distinct developmental states.

Here, we present a time‐resolved single‐nucleus transcriptomic landscape of 91 144 nuclei from the chicken pectoralis major across four postnatal developmental stages. Integrative analysis revealed dynamic changes in myogenic subpopulations, intercellular communication networks, and transcription factor activity underlying myofiber hypertrophy. We identify the cAMP‐responsive element modulator (CREM) as a brake on myofiber hypertrophy, acting through direct transcriptional activation of JUN to cooperatively sustain the expression of muscle growth inhibitors and repress myofiber hypertrophy. This work establishes a developmental single‐nucleus resource and defines a key checkpoint governing vertebrate myofiber hypertrophy.

## 2 Results

### 2.1 Divergent Myofiber Hypertrophy is Accompanied by Dynamic Remodeling of the Muscle Cellular Landscape

To characterize the phenotypic basis of divergent myofiber hypertrophy, we monitored growth performance and pectoralis major morphology in FG and SG chickens over a 42‐day postnatal period (Figure S1A). The divergence in pectoralis major weight (PMW) between FG and SG widened progressively with age, reaching a 10.1‐fold difference at D42 (Figure 1A,B). Myofiber cross‐sectional area (CSA) did not differ significantly between breeds at DOH but diverged markedly thereafter, with FG exhibiting a 4.7‐fold greater CSA than SG at D42 (Figure 1C,D), inversely correlating with myofiber density (Figure S1B). FG also possessed a significantly greater total number of myofibers than SG (Figure S1C), indicating that both fiber number and fiber size contribute to hypertrophic dominance in broilers. To explore the myogenic basis of this divergence, we examined the expression of key myogenic regulatory factors. *MYOD1*, a master driver of myogenic differentiation, was significantly upregulated in FG at D28 (Figure S1D), whereas *PAX7*, a marker of quiescent satellite cells, was markedly elevated in SG at the same timepoint (Figure S1E). This reciprocal expression pattern suggests that FG muscle sustains more active myogenic differentiation while SG muscle retains a greater proportion of undifferentiated progenitors, providing a transcriptional basis for the observed hypertrophic divergence.

**FIGURE 1:**
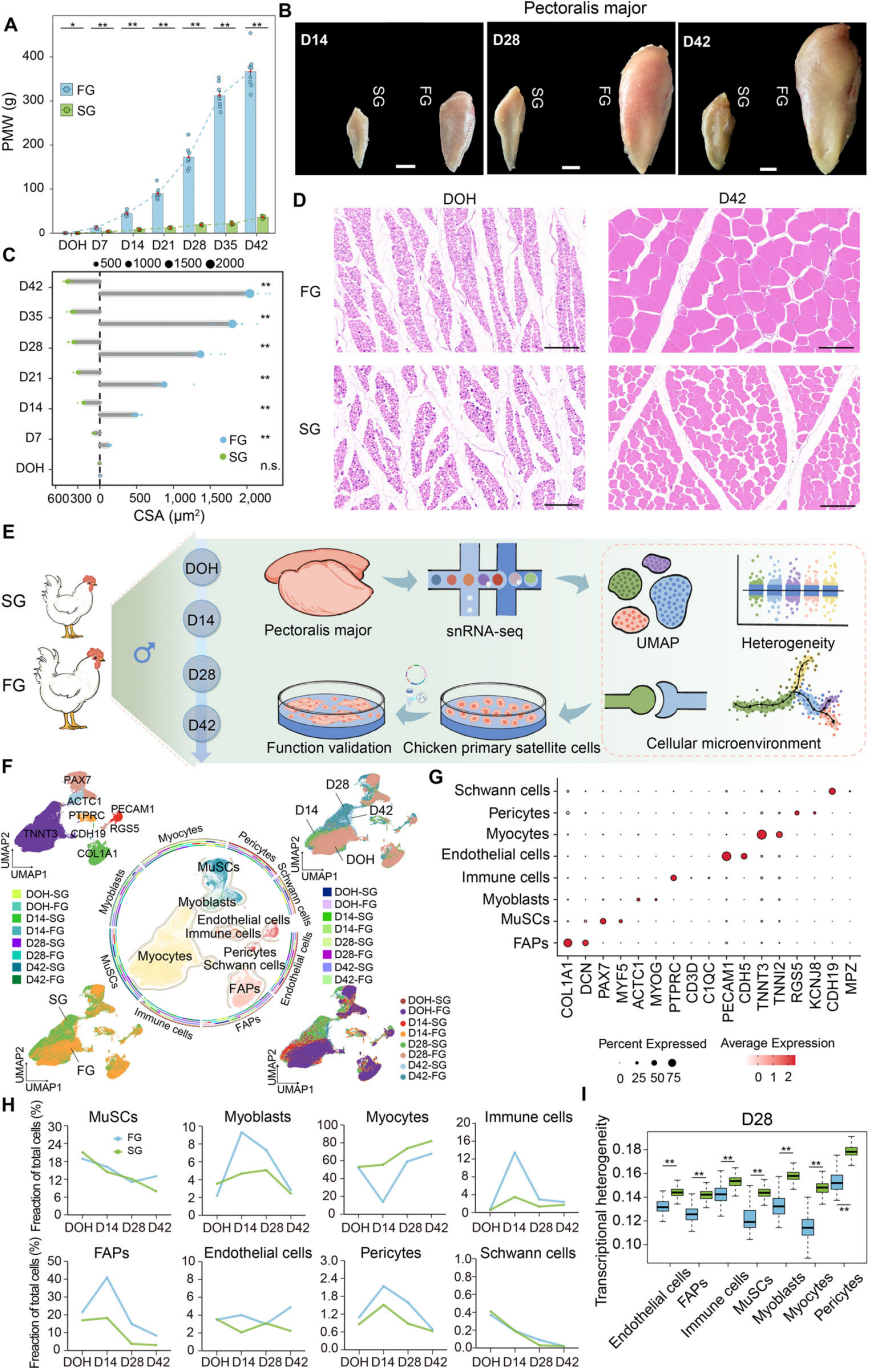
Postnatal myofiber hypertrophy is associated with dynamic remodeling of the pectoralis major cellular landscape. (A) Pectoralis major weight (PMW) of fast‐growing broilers (FG) and slow‐growing layers (SG) at day of hatch (DOH), day 7 (D7), D14, D21, D28, D35, and D42. Data are presented as mean ± SEM (*n* = 8). Statistical significance was determined by a two‐tailed unpaired Student's *t*‐test. (B) Representative images of pectoralis major muscle of FG and SG at D14, D28, and D42. Scale bar, 2 cm. (C) Dynamic changes in myofiber cross‐sectional area (CSA) from DOH to D42 in FG and SG. Large dots indicate the group means (*n* = 8). Statistical significance was determined by a two‐tailed unpaired Student's *t*‐test. (D) Representative hematoxylin and eosin (H&E) stained cross sections of pectoralis major muscle at DOH (Scale bar, 50 µm) and D42 (Scale bar, 100 µm). (E) Schematic workflow of the time course single nucleus RNA sequencing (snRNA‐seq) by 10× Genomics Chromium Controller and the downstream analytical pipeline. (F) Uniform manifold approximation and projection (UMAP) projection of 91 144 high‐quality nuclei from four time points (DOH, D14, D28, D42) revealing eight major cell populations. Top left: key marker genes for each cell population. Top right: distribution of cells across time points. Bottom left: distribution across FG and SG. Bottom right: distribution across sample individuals. (G) Dot plot showing top differentially expressed marker genes defining the eight cell populations. Dot size represents the percentage of nuclei expressing each gene; color intensity indicates the average expression level. (H) Dynamic changes in the proportion of each cell population in FG and SG from DOH to D42. (I) Transcriptional heterogeneity of each population between FG and SG at D28. The sample size for each group represents the number of nuclei within the respective cell population. Transcriptional heterogeneity scores between FG and SG groups were compared using a two‐sided Wilcoxon rank‐sum test. ^*^ *p* < 0.05; ^**^ *p* < 0.01; n.s. = not significant.

We then performed snRNA‐seq on pectoralis major muscle from FG and SG across four postnatal time points (Figure 1E). Following stringent quality control filtering, 91 144 high‐quality nuclei were retained for all downstream analyses (Table S1). Unbiased clustering identified eight major cell populations (Figure 1F). These populations were annotated based on marker gene expression: myocytes (*TNNT3*, *TNNI2*) [19], muscle satellite cells (MuSCs) (*PAX7*, *MYF5*) [20], myoblasts (*ACTC1*, *MYOG*) [21], immune cells (*PTPRC*, *CD3D*, *C1QC*) [19], fibro‐adipogenic progenitors (FAPs) (*COL1A1*, *DCN*) [19], endothelial cells (*PECAM1*, *CDH5*) [22], pericytes (*RGS5*, *KCNJ8*) [23], and schwann cells (*CDH19*, *MPZ*) [24] (Figure 1G).

Although the eight cell populations were conserved between FG and SG, their proportional composition showed pronounced breed‐associated dynamics across development (Figure 1H). MuSC proportions declined progressively in both groups yet remained higher in FG at D42 (13.00% vs. 7.94%). Myoblasts expanded substantially in FG at D14, reaching approximately twice the proportion observed in SG, indicative of accelerated myogenic commitment. In contrast, myocytes were approximately fourfold more abundant in SG at the same timepoint, consistent with earlier terminal differentiation. Notably, immune cells, FAPs, and pericytes accounted for persistently higher fractions in FG throughout development, with peak enrichment at D14 (immune cells: 13.45%; FAPs: 40.91%; pericytes: 2.14%), suggesting that the non‐myogenic niche is substantially more active in broilers during the critical window of myofiber hypertrophy. From D14 onward, SG exhibited progressively elevated transcriptional heterogeneity across all cell populations, becoming significantly greater than that of FG at all subsequent timepoints (Figure 1I and Figure S1F), indicating that FG muscle maintains a more transcriptionally coordinated state conducive to sustained myofiber hypertrophy. These findings establish that divergent myofiber hypertrophy between FG and SG is underpinned by breed‐associated differences in myogenic commitment, non‐myogenic niche composition, and transcriptional coordination, providing the cellular framework for deeper interrogation of the regulatory programs governing postnatal muscle growth.

### 2.2 Myogenic Subpopulation Dynamics Reveal Distinct Differentiation Trajectories Underlying Myofiber Hypertrophy

To resolve myogenic progression at higher resolution, MuSCs, myoblasts, and myocytes were merged and subclustered, revealing seven transcriptionally distinct cell subpopulations: quiescent MuSCs, proliferating MuSCs, differentiating MuSCs, myoblasts, *MYBPC1* ^+^ myocytes, *KLHL40* ^+^ myocytes, and *AK1* ^+^ myocytes (Figure 2A). Expression heatmap of the top 50 signature genes per subpopulation confirmed the transcriptional uniqueness of each subpopulation (Figure 2B and Table S2). Quiescent MuSCs were enriched for niche maintenance and basement membrane genes, including *PAX7* and *LAMA2*, while proliferating MuSCs showed prominent cell cycle and mitotic signatures. Translational machinery components and early differentiation factors were upregulated in differentiating MuSCs and myoblasts, whereas *MYBPC1* ^+^ myocytes and *KLHL40* ^+^ myocytes were characterized by contractile and structural programs. Notably, we identified a distinct myocyte subpopulation marked by *AK1* expression that uniquely co‐expresses genes encoding muscle cytoskeletal components (*TNNC1*, *TPM1*) [25], glycolytic enzymes (*LDHA*, *PGK2*) [26], and mitochondrial oxidative phosphorylation factors (*COX1*, *COX2*, *COX3*, *ATP5* family) [27, 28], revealing a previously unappreciated transcriptional state poised between differentiation commitment and functional maturation. These subpopulations recapitulate the full progression from stem cell quiescence to terminal differentiation, establishing the cellular framework for understanding myofiber hypertrophy.

**FIGURE 2:**
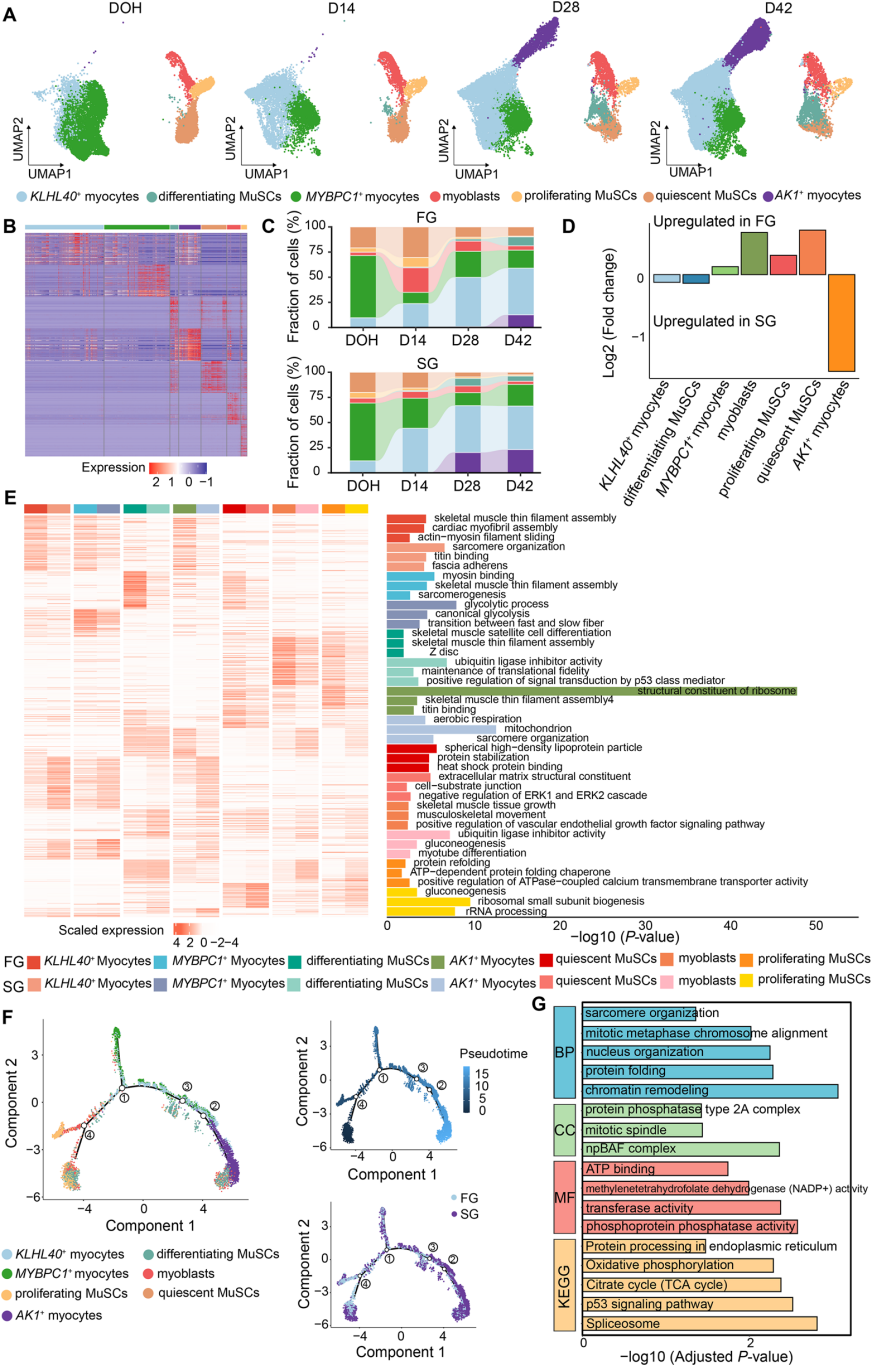
Myogenic subpopulation dynamics underlie divergent myofiber hypertrophy trajectories. (A) UMAP visualization of the integrated myogenic lineage (comprising MuSCs, myoblasts, and myocytes) resolved into seven transcriptionally distinct subpopulations across DOH to D42. (B) Expression of the top 50 signature genes for each subpopulation. Color intensity indicates average expression level. (C) Temporal dynamics of subpopulation proportions in FG and SG from DOH to D42. (D) Aggregate analysis across all time points showing fold change in subpopulation abundance between FG and SG. (E) Gene Ontology (GO) enrichment analysis of differentially expressed genes between FG and SG chickens within each myogenic subpopulation. (F) Pseudotime trajectory inference ordering myogenic cells along a continuous differentiation path, with branch points annotated. (G) GO and Kyoto Encyclopedia of Genes and Genomes (KEGG) pathway enrichment analysis of genes associated with branch point 2, as identified by branch expression analysis modeling (BEAM).

The relative abundance of myogenic subpopulations exhibited dynamic changes during post‐hatch development in both FG and SG. *MYBPC1* ^+^ myocytes showed a decreasing trend in relative representation from DOH to D42, while *KLHL40* ^+^ myocytes reciprocally increased, reflecting a conserved maturation trajectory (Figure 2C). In contrast, myoblasts displayed dynamic differences in their relative frequencies. Their proportion remained consistently low in SG throughout development (3.10%–6.67%), whereas in FG, myoblasts expanded substantially at D14 (24.51%) before declining to 10.20% by D28, indicating a transient but pronounced burst of myogenic commitment during the critical window of rapid muscle growth. Concurrently, *AK1* ^+^ myocytes were detected earlier in the SG dataset (first detected at D28) than in FG (first detected at D42) (Figure 2C), suggesting accelerated progression toward terminal differentiation in slow‐growing birds. Consistent with these dynamics, quiescent MuSCs, proliferating MuSCs, and myoblasts were significantly enriched in FG, whereas *AK1* ^+^ myocytes were markedly elevated in SG (Figure 2D). The transient increase in myoblast representation in FG at D14 and delayed detection of *AK1* ^+^ myocytes collectively suggest a model where sustained myogenic commitment, rather than early terminal differentiation, is the primary cellular mechanism driving myofiber hypertrophy in fast‐growing birds.

To understand the transcriptional programs underlying these divergent dynamics, we performed differential gene expression analysis across myogenic subpopulations (Figure S2A and Table S3). *TNNC1*, a key regulator of striated muscle contraction [29], was consistently upregulated in SG across six subpopulations, consistent with earlier contractile maturation. In contrast, FG showed elevated expression of genes involved in active myogenic differentiation and cytoskeletal organization, including *ACTC1* and *MUSTN1* in differentiating MuSCs and *AK1* ^+^ myocytes, and *CLU* and *HSP90AA1* across multiple subpopulations, suggesting enhanced proteostasis supporting continued myofiber growth. *RBM24* and *ZEB2*, established regulators of myogenic commitment and RNA splicing [30], were upregulated in FG myoblasts, further supporting a more robust differentiation trajectory in fast‐growing birds. Gene ontology (GO) enrichment analysis revealed that FG‐enriched genes were predominantly associated with myogenic differentiation, sarcomere assembly, and protein homeostasis, whereas SG‐enriched genes were strongly linked to glycolytic metabolism, mitochondrial respiration, and extracellular matrix organization (Figure 2E). These transcriptional signatures collectively indicate that FG muscle prioritizes anabolic and structural programs that support myofiber hypertrophy, whereas SG muscle is directed toward metabolic adaptation and matrix remodeling.

Pseudotime trajectory analysis placed quiescent and proliferating MuSCs at the origin of the myogenic progression, with cells diverging with cells branching at node 1 toward either *MYBPC1* ^+^ myocytes or a terminal path culminating in *KLHL40* ^+^ myocytes and *AK1* ^+^ myocytes (Figure 2F). *AK1* ^+^ myocytes appeared earlier along the pseudotime axis in SG than in FG (Figure S2B), consistent with the earlier onset of terminal differentiation in slow‐growing birds. Branch expression analysis modeling (BEAM) analysis at branch point 2 identified a transcriptional program primed for transcriptional divergence, with enriched terms implicating sarcomere assembly, chromatin remodeling, and mitotic regulation (Figure S2C). Kyoto Encyclopedia of Genes and Genomes (KEGG) pathways highlighted oxidative phosphorylation, TCA cycle, and p53 signaling, alongside spliceosome and endoplasmic reticulum protein processing, suggesting that metabolic state, proteostasis, and epigenetic control may jointly influence the fate choices between fetal and terminal myocyte trajectories (Figure 2G). These results are consistent with a model in which the earlier onset of terminal myocyte differentiation in SG is shaped by a convergence of metabolic and epigenetic programs that potentially accelerate transcriptional divergence away from the proliferative progenitor pool, thereby possibly limiting the myogenic capacity available to support sustained myofiber hypertrophy.

### 2.3 Non‐Myogenic Niche Cells Sustain Myofiber Hypertrophy Through Coordinated Intercellular Communication

To explore the potential roles of non‐myogenic niche cells in myofiber hypertrophy, we characterized FAP and immune cell subpopulations and mapped their intercellular communication networks within the postnatal muscle niche. FAPs were resolved into seven transcriptionally distinct subpopulations, including myocyte‐like FAPs, proliferating FAPs, matrix FAPs, metabolically active FAPs, *EDIL3* ^+^ FAPs, *GPC6* ^+^ FAPs, and adipocytes (Figure 3A and Figure S3A). Their relative abundance exhibited dynamic shifts during postnatal development, with metabolically active FAPs expanding dramatically in SG between D14 and D28 (1.54% to 47.10%), while remaining largely unchanged in FG (Figure S3B). Differential expression analysis across the four developmental timepoints revealed that breed‐specific transcriptional divergence was most pronounced at early stages (Table S4). Despite relatively few differentially expressed genes overall, matrix FAPs exhibited profound functional divergence between breeds (Figure 3B and Figure S3C–F). In FG, matrix FAPs adopted a pro‐regenerative state characterized by enhanced extracellular matrix remodeling, sustained *IGF1* expression at both D14 and D28 (Figure 3C), and activation of signaling programs supporting myoblast commitment and vascularization. Consistently, *IGF1* mRNA was significantly higher in FG pectoralis major throughout development (Figure 3D). In contrast, matrix FAPs in SG displayed a fibrogenic and anti‐angiogenic program, marked by sustained TGF‐β activity and suppression of myogenic cues, reinforcing a static, matrix‐preserving environment (Table S4). These findings suggest that matrix FAPs may act as key architects of a divergent myogenic microenvironment.

**FIGURE 3:**
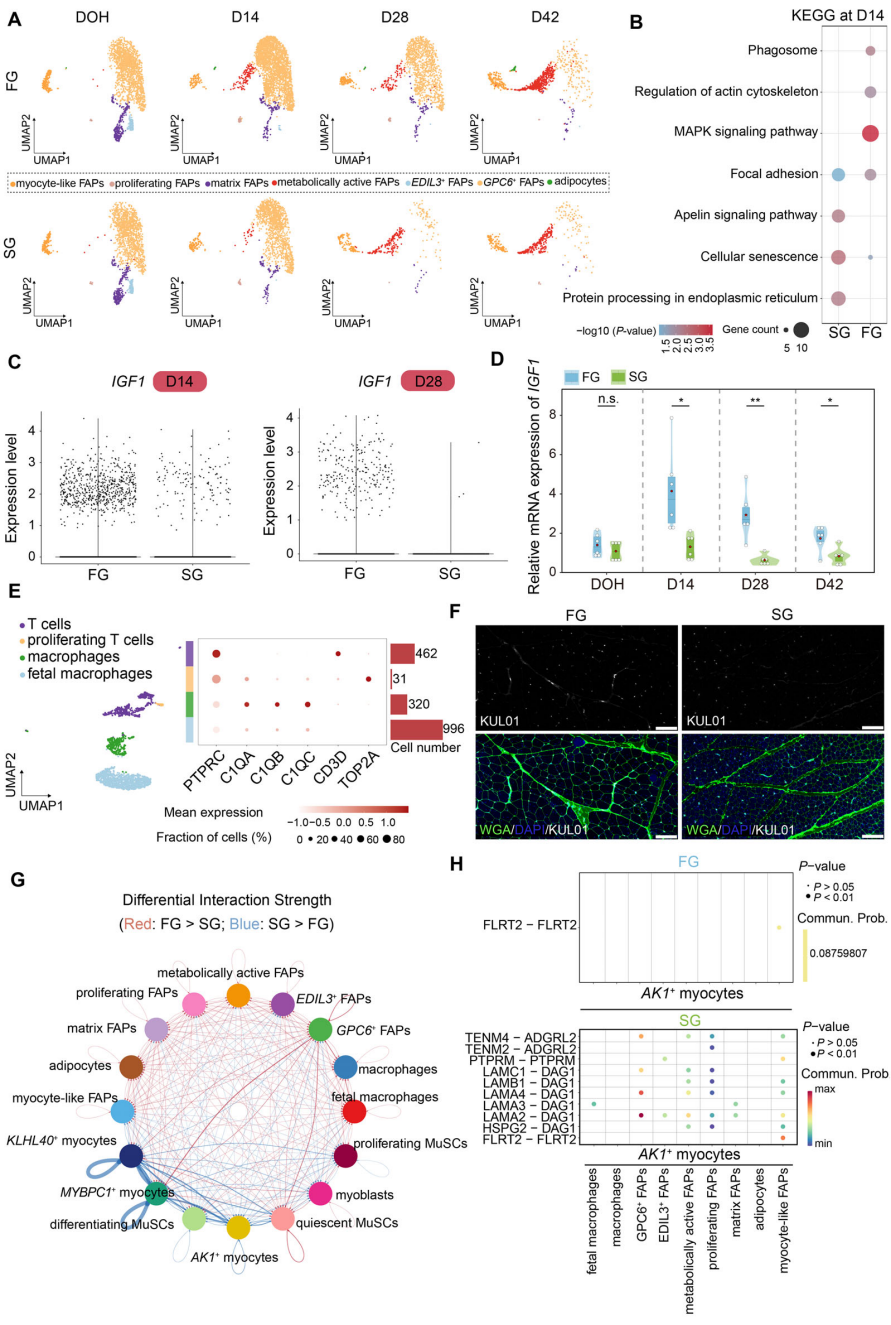
Coordinated intercellular communication between non‐myogenic niche cells and myogenic progenitors sustains myofiber hypertrophy. (A) Functional heterogeneity of seven subpopulations of fibro‐adipogenic progenitors (FAPs) across breeds and developmental stages. (B) KEGG pathway enrichment analysis of differentially expressed genes in matrix FAPs at D14. (C) Expression levels of *IGF1* in FAPs between FG and SG. (D) Real‐time quantitative PCR (RT‐qPCR) analysis of *IGF1* mRNA levels in pectoralis major of FG and SG at DOH, D14, D28, and D42. Red dots indicate the group means (*n* = 6). Statistical significance was determined by a two‐tailed unpaired Student's *t*‐test. ^*^ *p* < 0.05; ^**^ *p* < 0.01; n.s. = not significant. (E) Identification of immune cell subpopulations. The right panel shows nuclear abundance and top marker genes for four subpopulations. (F) Immunofluorescence staining of mononuclear macrophages in pectoralis major at D28 using anti‐KUL01 antibody (white), with WGA labeling myofiber cell membranes (green) and DAPI marking nuclei (blue). Scale bar, 100 µm. (G) Network map of interaction strength. Red edges denote stronger signaling in FG; blue edges denote stronger signaling in SG. (H) Ligand‐receptor pairs through which FAP subpopulations, macrophages, and fetal macrophages signal to *AK1* ^+^ myocytes in FG and SG.

The immune compartment was resolved into four distinct subpopulations: fetal macrophages, macrophages, proliferating T cells, and T cells (Figure 3E). Fetal macrophages dominated the immune landscape at DOH in both breeds but declined with age more rapidly in SG, such that by D28 they accounted for only 3.45% of immune cells in SG compared to 55.74% in FG. Concurrently, conventional macrophages expanded more rapidly in SG (36.21% vs. 13.66% at D28) (Figure S4A,B). Immunofluorescence staining of D28 pectoralis major muscle using the monocyte/macrophage marker KUL01 [31] confirmed a greater number of KUL01^+^ cells in FG (Figure 3F). Differential expression and GO enrichment analyses revealed that macrophages in FG were enriched for IGF receptor signaling, collagen catabolism, and phagosome maturation programs (Figure S4C–E and Table S5), consistent with potential active roles in extracellular matrix remodeling and growth factor delivery that facilitate muscle accretion.

To understand how these non‐myogenic cells might interact with myogenic populations, we mapped predicted intercellular communication networks among myogenic subpopulations, FAP subpopulations, and macrophages. FG exhibited greater overall crosstalk strength and number of interactions than SG, with interactions between fetal macrophages and myogenic progenitors, as well as between myogenic progenitors and myocyte‐like FAPs, substantially enhanced in FG (Figure S5A,B). Fetal macrophages in FG communicated with nearly all subpopulations, whereas those in SG interacted with only five (Figure S5C,D). Similarly, macrophages in FG actively engaged with differentiating MuSCs, myocyte‐like FAPs, and adipocytes, while SG macrophages maintained interactions solely with fetal macrophages (Figure S5E,F). CellChat predicts a paracrine IGF1‐IGF1R signaling axis. At D28, myocyte‐like FAPs served as the primary source of IGF1, signaling to quiescent, proliferating, and differentiating MuSCs via IGF1R, suggesting a paracrine axis that may support robust muscle growth in FG (Figure S5G). In contrast, *AK1* ^+^ myocytes in SG engaged in more numerous interactions, with the strongest incoming signals originating from GPC6^+^ FAPs via LAMA2‐DAG1, LAMA4‐DAG1, and TENM4‐ADGRL2 (Figures 3G,H and S5H,I,S6), hypothesizing that terminally differentiated myocytes in SG receive niche signals that may reinforce, rather than reverse, their committed state. Collectively, these inferred communication patterns suggest that enhanced niche‐myofiber crosstalk in FG, driven by pro‐growth IGF signaling from myocyte‐like FAPs and the broad communicative activity of fetal macrophages, may contribute to a permissive microenvironment that sustains myofiber hypertrophy, whereas the elevated intercellular connectivity of *AK1* ^+^ myocytes in SG reflects a fundamentally distinct niche state associated with terminal differentiation.

### 2.4 Integrative Transcriptomic Analysis Identifies CREM as a Brake on Myofiber Hypertrophy

Given that *AK1* ^+^ myocytes represent a terminally differentiated subpopulation enriched in SG, we sought to identify the transcriptional regulators governing this terminal myogenic fate. To this end, we performed through hierarchical deterministic weighted gene co‐expression network analysis (hdWGCNA) on the three myogenic lineage subpopulations (*MYBPC1* ^+^ myocytes, *KLHL40* ^+^ myocytes, and *AK1* ^+^ myocytes), with 14 selected as the optimal soft threshold (Figure S7A,B and Table S6). Seven co‐expression modules with highly correlated gene members were identified (Figure 4A and Table S7), and stemness scoring revealed that *AK1* ^+^ myocytes exhibited the lowest stemness score across all myogenic subpopulations (Figure 4B). Genes of the green, yellow, and red modules were highly expressed in the *AK1* ^+^ myocytes (Figure S7C), with the green module was enriched for mitochondrial and ribosomal genes such as *FTH1*, *COX2*, and *RPS17*, the yellow module was dominated by fast‐twitch contractile and glycolytic genes including *TNNC1*, *TPM1*, and *LDHA*, and the red module was primarily composed of heat shock proteins and sarcomere‐associated chaperones (*HSP90AA1*, *BAG3*, and *KLHL40*). Notably, the red module showed the strongest negative correlation with stemness among all modules (Figure 4C and Table S8), and further functional enrichment analysis revealed significant enrichment of MAPK and adrenergic signaling pathways within this module (Figure 4D), implicating it as a core transcriptional program driving terminal myocyte fate.

**FIGURE 4:**
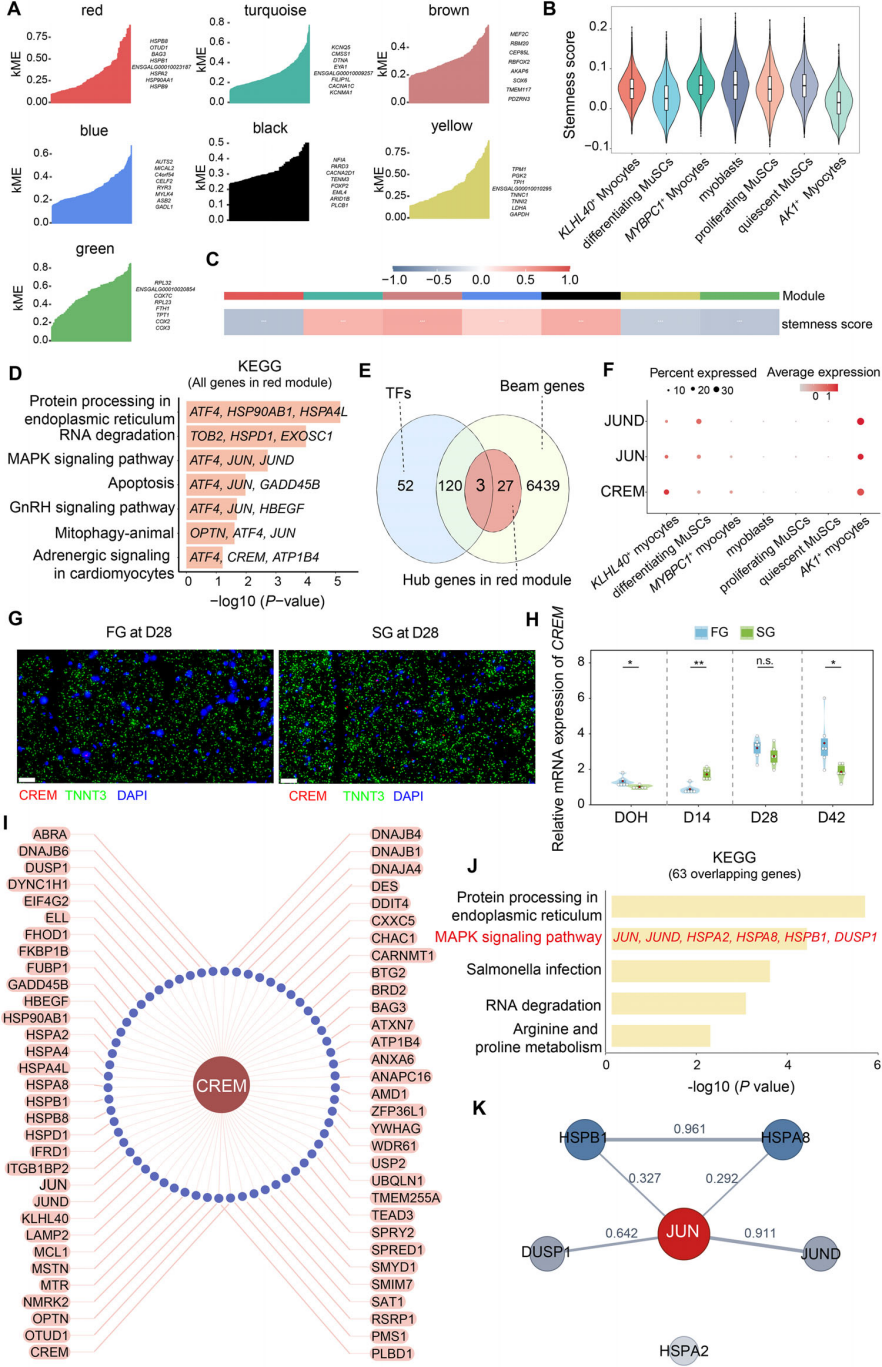
Integrative analysis identifies CREM as a master transcriptional repressor of myofiber hypertrophy. (A) Hierarchical deterministic weighted gene co‐expression network analysis (hdWGCNA) linking *MYBPC1* ^+^ myocytes, *KLHL40* ^+^ myocytes, and *AK1* ^+^ myocytes to a gene set related to stem cell differentiation (GO:0048863), yielding seven co‐expression modules. (B, C) Stemness scores across myogenic subpopulations (B) and seven modules (C). (D) KEGG enrichment of all genes in the red module. (E) Integrative prioritization of key transcription factors (TFs) by overlapping three gene sets (predicted transcription factors, BEAM‐identified genes at branch point 2, and hub genes of the red co‐expression module). (F) Expression of three overlapping TFs (CREM, JUN, JUND) across myogenic subpopulations. Dot size represents the percentage of expressing cells; color intensity indicates average expression level. (G) RNA in situ hybridization of *CREM* (red) in pectoralis major of FG and SG at D28, co‐stained with *TNNT3* (green, myofibers) and DAPI (blue, nuclei). Scale bar, 20 µm. (H) RT‐qPCR quantification of *CREM* mRNA in pectoralis major muscles of FG and SG at DOH, D14, D28, and D42. Red dots indicate the group means (*n* = 6). Statistical significance was determined by a two‐tailed unpaired Student's *t*‐test. ^*^ *p* < 0.05; ^**^ *p* < 0.01; n.s. = not significant. (I) Interaction network of 63 overlapping genes between the red module and predicted CREM targets. (J) KEGG enrichment of the 63 overlapping genes. (K) Protein‐protein interaction (PPI) network of genes enriched in the MAPK signaling pathway.

To pinpoint the key transcription factors governing this program, we integrated three gene sets: transcription factors predicted by pySCENIC, differentiation‐associated genes identified at branch point 2 by branched expression analysis modeling (BEAM) analysis, and hub genes of the red co‐expression module (Figure 4E and Table S9). This intersection yielded three high‐confidence transcription factors as candidate regulators of this terminal myogenic state, including CREM, JUN, and JUND. All three transcription factors were highly expressed in *AK1* ^+^ myocytes (Figure 4F and Figure S7D) and showed elevated expression in SG myocyte subpopulations at D28 (Figure S7E). Among the three, CREM exhibited the strongest correlation with the red module (Table S10), nominating it as the most probable master regulator of this terminal co‐expression program. To validate CREM expression at the tissue level, RNA in situ hybridization at D28 revealed no significant difference in overall *CREM* signal intensity between FG and SG (Figure 4G and Figure S7F), indicating that the snRNA‐seq finding reflects cell‐type‐specific rather than bulk transcriptional differences. Across four developmental timepoints, bulk *CREM* mRNA expression in the pectoralis major was most divergent at D14, where levels were significantly higher in SG than in FG, diminishing by D28, and reversing at D42with significantly elevated expression in FG (Figure 4H).

To identify the key downstream targets through which CREM exerts its regulatory effects, we predicted CREM target genes and intersected the resulting gene set with all members of the red co‐expression module, reasoning that genes both directly regulated by CREM and co‐expressed within the same network are most likely to be functionally relevant effectors (Figure 4I and Figure S7G and Table S11). This approach yielded 63 candidate genes, which showed significant KEGG enrichment in the MAPK signaling pathway (Figure 4J and Table S12), a well‐established cascade governing myogenic differentiation. Six MAPK pathway genes (*JUN*, *JUND*, *DUSP1*, *HSPB1*, *HSPA8*, *HSPA2*) were extracted for protein‐protein interaction network analysis, which identified *JUN* as the hub gene with the highest connectivity (Figure 4K). Taken together, these integrative analyses converge on the CREM/JUN transcriptional axis as a central regulatory node of terminal myogenic fate, with CREM functioning as a master regulator of the red co‐expression module and *JUN* serving as its key downstream effector, collectively defining a transcriptional program that restrains myogenic progenitor activity and limits myofiber hypertrophy.

### 2.5 *CREM* Knockdown Promotes Myogenic Differentiation and Myotube Formation in Primary Satellite Cells

To investigate the functional role of *CREM* in myogenic differentiation, chicken primary satellite cells (CPSCs) were isolated and subjected to time‐course profiling across proliferation (GM) and differentiation stages (DM1‐DM3). *CREM* expression was elevated during the proliferative phase and early differentiation (GM and DM1), declining progressively as differentiation advanced through DM2 and DM3 (Figure 5A), placing its peak activity in the early differentiation phases. Canonical myogenic regulatory factors displayed two temporally distinct expression patterns. *MYOD1* and *MYF5* peaked at DM2 before declining, consistent with roles in early differentiation commitment, whereas *MYOG*, *MYHC*, *TMEM8C*, and *MYMX* increased progressively from GM to DM3 (Figure S8A–C). This trajectory inversely mirroring that of *CREM*, suggestive of a functional antagonism between these factors and the late myogenic program.

**FIGURE 5:**
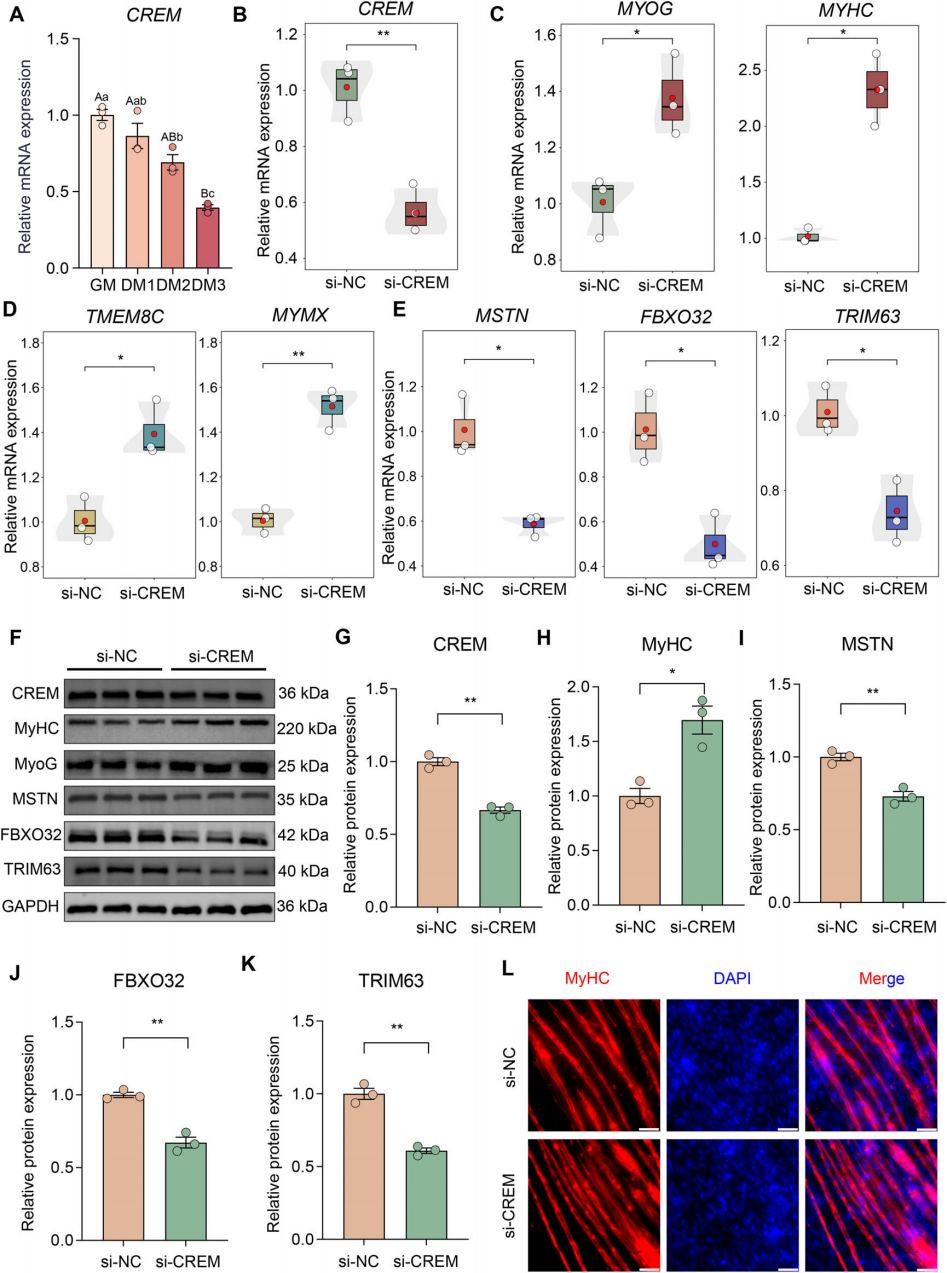
CREM suppresses myogenic differentiation in primary satellite cells. (A) mRNA expression of *CREM* in chicken primary satellite cells (CPSCs) across GM, DM1, DM2, and DM3 stages. Data are presented as mean ± SEM (*n* = 3). (B) mRNA expression of *CREM* in si‐NC and si‐CREM groups. Red dots indicate the group means (*n* = 3). (C) mRNA expression of *MYOG* and *MYHC* in si‐NC and si‐CREM groups. Red dots indicate the group means (*n* = 3). (D) mRNA expression of *TMEM8C* and *MYMX* in si‐NC and si‐CREM groups. Red dots indicate the group means (*n* = 3). (E) mRNA expression of *MSTN*, *FBXO32*, and *TRIM63* in si‐NC and si‐CREM groups. Red dots indicate the group means (*n* = 3). (F) Western blot images of CREM, MSTN, FBXO32, and TRIM63 in si‐NC and si‐CREM groups (*n* = 3). (G–K) Quantification of protein expression levels of CREM (G), MyHC (H), MSTN (I), FBXO32 (J), and TRIM63 (K). Data are presented as mean ± SEM (*n* = 3). (L) Representative immunofluorescence images of MyHC‐stained myotubes in si‐NC and si‐CREM groups. Scale bar, 100 µm. Data are presented as mean ± SEM (*n* = 3). Statistical significance was determined by a two‐tailed unpaired Student's *t*‐test, and comparisons among multiple groups were assessed using one‐way analysis of variance followed by Tukey's honest significant difference post hoc test. ^*^ *p* < 0.05; ^**^ *p* < 0.01; n.s. = not significant; different lowercase and uppercase letters indicate significant (*p* < 0.05) and highly significant (*p* < 0.01) differences, respectively; identical letters indicate no significant difference (*p* > 0.05).

*CREM* was silenced in CPSCs via siRNA, with si‐CREM‐1 selected for subsequent experiments based on highest knockdown efficiency among three candidates (Figure 5B and Figure S8D). CREM depletion had no significant effect on the mRNA expression of *MYOD1* or *MYF5* (Figure S8E,F), indicating that CREM does not interfere with early myogenic commitment. In contrast, the late‐stage differentiation drivers *MYOG*, *MYHC*, *TMEM8C*, and *MYMX* were markedly upregulated upon CREM knockdown (Figure 5C,D), demonstrating that CREM selectively restrains the late myogenic program. Concurrently, the expression of established negative regulators of muscle development, including *MSTN*, *FBXO32*, and *TRIM63*, was significantly downregulated at the mRNA level in si‐CREM cells (Figure 5E), suggesting that CREM normally sustains an inhibitory regulatory network to limit myogenic progression. At the protein level, CREM knockdown significantly increased the expression of MyHC and MyoG (Figure 5F–H and Figure S8G), while markedly reducing the abundance of MSTN, FBXO32, and TRIM63 (Figure 5I–K). Furthermore, CREM‐depleted cells exhibited a marked increase in relative myotube area and diameter (Figure 5L and Figure S8H), demonstrating that *CREM* knockdown is sufficient to promote myotube formation. These findings establish CREM as a negative regulator of myogenic differentiation in CPSCs, operating by sustaining the expression of muscle growth inhibitors MSTN, FBXO32, and TRIM63 and selectively suppressing the late‐stage myogenic transcriptional program, with its depletion sufficient to enhance myotube formation.

### 2.6 *CREM* Transcriptionally Activates *JUN* to Cooperatively Suppress Myogenic Differentiation

Prior transcriptomic analysis nominated *JUN* as a principal downstream target of *CREM*. To determine whether *CREM* exerts its inhibitory effects on myogenesis through direct transcriptional control of *JUN*, we first characterized the expression dynamics of *JUN* across the proliferative and differentiation stages of CPSCs. *JUN* expression declined progressively during differentiation, mirroring the temporal profile of *CREM* (Figure 6A). Then, we investigated the physical and functional interaction between *CREM* and the *JUN* promoter. A conserved *CREM*‐binding site was identified within the *JUN* promoter region across human, mouse, and chicken (Figure 6B and Figure S9A), suggesting evolutionary conservation of this regulatory interaction. Overexpression of *CREM* significantly elevated *JUN* mRNA levels (Figure 6C), while dual‐luciferase reporter assays demonstrated that *CREM* specifically drives *JUN* transcription through this binding site, an effect that was abolished upon its mutation (Figure 6D). Direct promoter occupancy was further confirmed by ChIP‐PCR (Figure 6E), collectively establishing CREM as a direct transcriptional activator of *JUN* in CPSCs.

**FIGURE 6:**
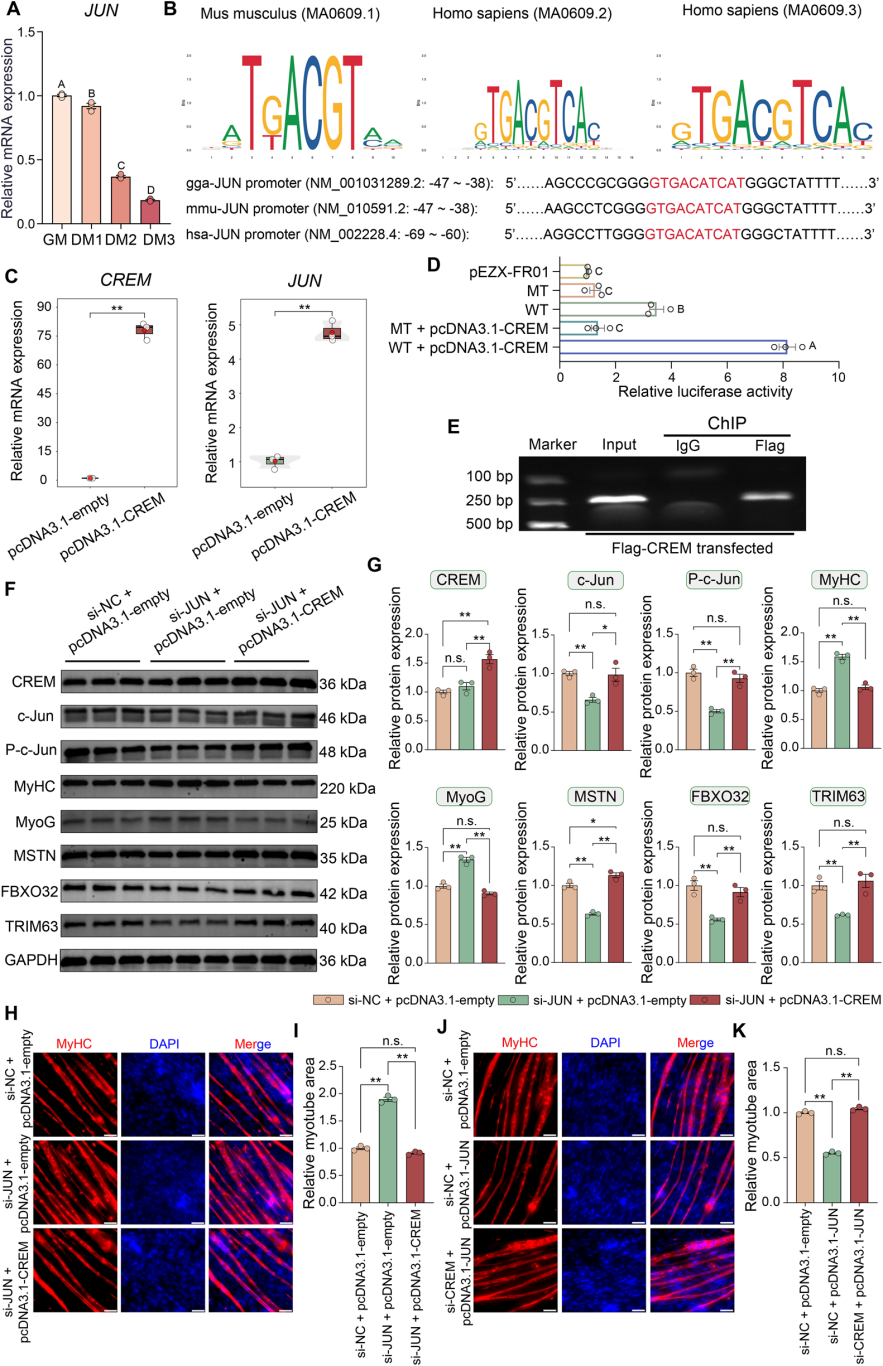
CREM directly activates JUN transcription to cooperatively repress myogenic differentiation. (A) mRNA expression of *JUN* in CPSCs across GM, DM1, DM2, and DM3 stages. Data are presented as mean ± SEM (*n* = 3). (B) Schematic illustration of the conserved CREM‐binding motif identified within the *JUN* promoter across human, mouse, and chicken. (C) mRNA expression of *CREM* and *JUN* following transfection with pcDNA3.1‐CREM or pcDNA3.1‐Empty. Red dots indicate the group means (*n* = 3). (D) Relative luciferase activity of wild‐type and mutant *JUN* promoter reporter constructs in the presence or absence of pcDNA3.1‐CREM. Data are presented as mean ± SEM (*n* = 3). (E) ChIP‐PCR validation of *CREM* occupancy at the *JUN* promoter in CPSCs. (F) Western blot images of CREM, c‐Jun, P‐c‐Jun, MyHC, MyoG, MSTN, FBXO32, and TRIM63 in si‐NC + pcDNA3.1‐Empty, si‐JUN + pcDNA3.1‐Empty, and si‐JUN + pcDNA3.1‐CREM groups. (G) Quantification of protein expression levels of CREM, c‐Jun, P‐c‐Jun, MyHC, MyoG, MSTN, FBXO32, and TRIM63 across the three groups. Data are presented as mean ± SEM (*n* = 3). (H) Representative immunofluorescence images of MyHC‐stained myotubes across the three groups. Scale bar, 100 µm. (I) Quantification of relative myotube area across the three groups. Data are presented as mean ± SEM (*n* = 3). (J) Representative immunofluorescence images of MyHC‐stained myotubes in si‐NC + pcDNA3.1‐Empty, si‐NC + pcDNA3.1‐JUN, and si‐CREM + pcDNA3.1‐JUN groups. Scale bar, 100 µm. (K) Quantification of relative myotube area across the three groups. Data are presented as mean ± SEM (*n* = 3). Data are presented as mean ± SEM. Comparisons between two groups were assessed using a two‐tailed unpaired Student's *t* test, and comparisons among three or multiple groups were assessed using one‐way analysis of variance followed by Tukey's honest significant difference post hoc test. ^*^ *p* < 0.05; ^**^ *p* < 0.01; n.s. = not significant; different lowercase and uppercase letters indicate significant (*p* < 0.05) and highly significant (*p* < 0.01) differences, respectively; identical letters indicate no significant difference (*p* > 0.05).

To determine whether the suppressive effects of CREM and JUN on myogenesis are mutually dependent rather than independent, bidirectional rescue experiments were performed in CPSCs. When *JUN* was knocked down, myotube formation was significantly enhanced, and the expression of negative muscle regulatory factors (FBXO32 and TRIM63) was markedly reduced, and these effects were fully reversed by CREM overexpression (Figure 6F–I and Figure S9B,C), demonstrating that the pro‐differentiation consequence of *JUN* loss requires concurrent CREM activity. In the reciprocal experiment, *JUN* overexpression suppressed myotube formation and upregulated the same inhibitory regulators, effects that were completely rescued by CREM knockdown (Figure 6J,K and Figure S9D–F). Notably, the terminal differentiation markers MyHC and MyoG remained largely unaffected across both rescue paradigms, whereas MSTN, FBXO32, and TRIM63 responded consistently and bidirectionally, pinpointing this inhibitory network as the primary effector of CREM/JUN cooperative action. Collectively, CREM and JUN function as cooperative transcriptional repressors of myogenesis.

### 2.7 Lentiviral‐Mediated *CREM* Silencing Promotes Myofiber Hypertrophy In Vivo

To validate the regulatory role of *CREM* in pectoralis muscle development in vivo, lentiviral vectors encoding CREM‐targeting siRNA (LV‐si‐CREM) or a negative control (LV‐si‐NC) were injected directly into the pectoralis muscle of healthy male broilers at D7, with a booster injection at D14, and tissues were harvested at D21 (Figure 7A). Successful knockdown was confirmed by a significant reduction in *CREM* mRNA expression in the pectoralis muscle of LV‐si‐CREM birds relative to controls (Figure 7B). Although body weight did not differ significantly between groups at D7 or D21 (Figure S9G), LV‐si‐CREM birds exhibited 29.96% higher PMW than the controls (63.50 g vs. 48.86 g), and 20.50% higher pectoralis major percent (PMP) (12.10% vs. 10.04%) at D21, demonstrating a direct promotion effect of CREM depletion on muscle mass accumulation (Figure 7C–E).

**FIGURE 7:**
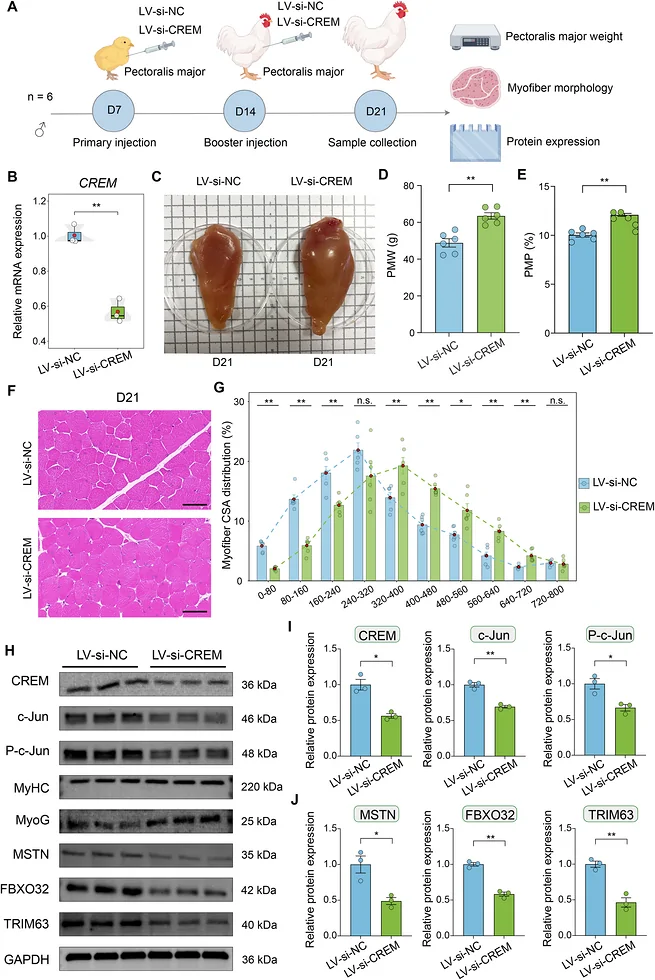
Lentiviral‐mediated CREM silencing promotes myofiber hypertrophy in vivo. (A) Schematic diagram of the lentiviral injection protocol. Lentiviral vectors were injected into the pectoralis major of 7‐day‐old male small white‐feather broilers from a developing line, with a booster injection at D14, and tissues were harvested at D21. Parts of this figure were created using materials from Figdraw. (B) mRNA expression of *CREM* in the pectoralis major of LV‐si‐NC and LV‐si‐CREM birds. Red dots indicate the group means (*n* = 3). (C) Representative photographs of the pectoralis major from LV‐si‐NC and LV‐si‐CREM birds at D21. (D) PMW of LV‐si‐NC and LV‐si‐CREM birds at D21. Data are presented as mean ± SEM (*n* = 6). (E) Pectoralis major percent (PMP) of LV‐si‐NC and LV‐si‐CREM birds at D21. Data are presented as mean ± SEM (*n* = 6). (F) Representative H&E‐stained cross‐sections of pectoralis major muscle fibers from LV‐si‐NC and LV‐si‐CREM birds at D21. Scale bar, 50 µm. (G) Frequency distribution of muscle fiber CSA in LV‐si‐NC and LV‐si‐CREM birds at D21. Data are presented as mean ± SEM (*n* = 6). (H) Western blot images of CREM, c‐Jun, P‐c‐Jun, MyHC, MyoG, MSTN, FBXO32, and TRIM63 in LV‐si‐NC and LV‐si‐CREM birds. (I) Quantification of protein expression levels of CREM, c‐Jun, and P‐c‐Jun. Red dots indicate the group means (*n* = 3). (J) Quantification of protein expression levels of MSTN, FBXO32, and TRIM63. Red dots indicate the group means (*n* = 3). Data are presented as mean ± SEM. Statistical significance was determined by a two‐tailed unpaired Student's *t*‐test. ^*^ *p* < 0.05; ^**^ *p* < 0.01; n.s. = not significant.

At the histological level, H&E‐stained cross‐sections revealed visibly larger myofibers in the pectoralis muscle of LV‐si‐CREM birds, with quantitative analysis confirming a markedly greater proportion of large‐CSA myofibers and a distribution shifted toward higher cross‐sectional area ranges (Figure 7F,G). Following in vivo knockdown of CREM, the mRNA expression level of *JUN* was significantly decreased (Figure S9H). Furthermore, the protein abundance of CREM was significantly reduced in LV‐si‐CREM birds, accompanied by concordant decreases in c‐Jun and P‐c‐Jun (Figure 7H,I). The muscle growth inhibitors and atrophy‐related E3 ubiquitin ligases MSTN, FBXO32, and TRIM63 were all highly significantly downregulated at the protein level (Figure 7J), consistent with the in vitro findings and demonstrating that CREM/JUN‐mediated maintenance of this inhibitory network operates at the tissue level. MyHC protein levels were significantly decreased, whereas no significant difference in MyoG protein levels was observed between groups (Figure S9I). These in vivo findings confirm that attenuation of CREM is sufficient to drive myofiber hypertrophy and increase muscle mass in the postnatal pectoralis major, establishing CREM as a transcriptional brake on myofiber hypertrophy.

## 3 Discussion

In this study, we present a time‐resolved single‐nucleus transcriptomic profiling of the chicken pectoralis major across four postnatal developmental stages, capturing 91 144 high‐quality nuclei. By delineating the transcriptional programs underlying myofiber hypertrophy, we uncovered dynamic interactions among myogenic subpopulations, FAPs, and immune cells that collectively sustain muscle growth. Integrative transcription factor analysis identified CREM as a previously unrecognized transcriptional brake on myofiber hypertrophy, a role mechanistically substantiated through its direct transcriptional activation of *JUN* and cooperative suppression of a muscle growth inhibitor, and further confirmed by lentiviral‐mediated silencing in vivo.

The pectoralis major of FG contained nearly twice the number of myofibers compared to that of SG, with post‐hatch myofiber hypertrophy occurring at a significantly accelerated rate, consistent with our previous observations [32]. Unlike embryonic myogenesis, which is characterized by active myoblast fusion, myofiber formation, and extensive extracellular matrix remodeling [33], our data reveal that the cellular composition of the pectoralis major remains largely stable throughout post‐hatch development, which is consistent with snRNA‐seq data from embryonic chicken leg muscle [34]. This stability suggests that breed‐specific differences in postnatal muscle growth are governed not by alterations in cell population identity, but by dynamic regulation of the microenvironment. Pseudotime trajectory analysis effectively recapitulated the canonical differentiation pathway from satellite cells through myoblasts to mature myocytes [35, 36, 37], with positive correlations between satellite cells, FAPs, and immune cells increasing at D28, suggesting that stromal and immune components collaboratively facilitate myofiber hypertrophy during advanced stages of growth [38].

We identified a novel terminally differentiated myocyte subpopulation (*AK1* ^+^ myocytes) that emerged earlier and was markedly more abundant in SG than in FG birds. This subpopulation co‐expressed canonical myofiber genes (*TNNC1*, *TNNI2*, and *TPM1*) [39], along with markers associated with glycolysis and oxidative phosphorylation (*GAPDH*, *EEF1A2*, and *RPL3L*) [40]. The premature appearance of this terminal state in SG suggests that slow‐growing birds may undergo accelerated exhaustion of the satellite cell pool, thereby constraining postnatal myofiber hypertrophy. Conversely, FG satellite cells appear to maintain a higher stemness and myogenic potential, enabling more effective fusion into developing myofibers and supporting accelerated muscle growth. This aligns with the prevailing concept that the balance between progenitor self‐renewal and terminal differentiation dictates the long‐term growth capacity of skeletal muscle [41]. We also identified a *MYBPC1* ^+^ myocyte subpopulation expressing embryonic myogenesis markers (*MYBPC1*, *CYP7B1*, *FGFRL1*, and *CACNA1C*) [42], likely derived from myofibers formed prior to hatching and retaining a residual embryonic transcriptional signature.

FAPs are recognized to have three primary differentiation potentials: fibrogenic, myogenic, and adipogenic [43, 44]. Consistent with previous research, all three lineages were mapped within the chicken FAP compartment, with fibrogenic subpopulations predominating. During early post‐hatch development, connective tissue expansion surrounds myofibers to support skeletal muscle homeostasis, but this compartment becomes increasingly fibrotic as myofibers undergo accelerated hypertrophy in FG [45]. In FG, matrix FAPs adopted a pro‐regenerative state characterized by enhanced ECM remodeling and sustained IGF1 expression, fostering a pro‐anabolic microenvironment. In contrast, SG matrix FAPs displayed a fibrogenic and anti‐angiogenic program marked by TGF‐β activity, which may restrict further muscle expansion. Similarly, fetal‐derived macrophages, known to potently support postnatal myogenesis [46], had transcriptional profiles consistent with enhanced signaling output, as confirmed by immunofluorescence staining. Genes differentially expressed in FG macrophages were predominantly enriched in pathways associated with macrophage activation, protein metabolism, and IGF signaling [47], indicating their potential contribution to a pro‐anabolic microenvironment.

Cell‐cell communication analysis revealed dynamic differences in ligand‐receptor interaction networks associated with divergent muscle growth. IGF signaling, a well‐established positive regulator of skeletal muscle growth and protein synthesis [48], showed robust predicted activity in FG, with myocyte‐like FAPs identified as a potential primary source of IGF1 at D28, predicted to signal to MuSCs via IGF1R. This paracrine axis was further supported by consistently elevated IGF1 mRNA levels in FG pectoralis major throughout development [49]. In SG, GPC6^+^ FAPs were predicted to engage in extensive crosstalk with *AK1* ^+^ myocytes through laminin‐DAG1 interactions (LAMA2‐DAG1, LAMA4‐DAG1, LAMC1‐DAG1) and HSPG2‐DAG1 signaling [50, 51], interactions previously implicated in satellite cell aging and stemness depletion [52]; this niche interaction may actively accelerate the premature terminal differentiation and limit the regenerative capacity in SG. D14 and D28 emerged as critical windows during which the muscle microenvironment diverges most substantially between breeds, with sustained IGF signaling and intensified FAP‐macrophage‐myogenic crosstalk potentially contributing to rapid muscle accretion in FG. It is important to note that these intercellular communication networks are inferred from transcriptomic data, and future functional validation through co‐culture experiments or targeted cytokine modulation studies are required to establish causal relationships

Furthermore, in‐depth data analyses and functional experiments identified CREM as the transcriptional repressor of myogenic differentiation in chicken and elucidated its downstream mechanism through direct regulation of *JUN*. CREM is a highly conserved basic leucine zipper transcription factor that modulates target gene expression by binding to cAMP response elements within promoter regions [53], with its extensive alternative splicing generating isoforms that act as either activators or repressors [53]. In mammals, CREM plays distinct roles across different tissues. For instance, it acts as a critical marker and activator driving spermatid differentiation and increased sperm count [54]. Conversely, in the mammalian heart, the aberrant expression of repressor isoforms of CREM (CREM‐IbΔC‐X) induces pro‐arrhythmic alterations, suggesting that CRE‐dependent transcriptional repression contributes to cardiac pathology [55]. In contrast to its activating role in spermatogenesis, our study reveals that CREM functions primarily as a brake that restrains myofiber hypertrophy in avian skeletal muscle. This inhibitory function is consistent with the repressive mechanism observed in cardiac tissues [55] and is likely mediated by specific repressor isoforms or co‐repressor complexes. Here, we identified JUN as a key downstream effector, forming a novel CREM‐JUN axis that maintains the expression of muscle growth inhibitors. While CREM has been implicated in avian inner ear regeneration [56] and cardiac oxidative stress [57], this work is the first to demonstrate that CREM acts as a negative regulator of skeletal myogenesis in avian species and to identify *JUN* as its direct downstream effector. In our in vitro assays, CREM knockdown achieved approximately 50% mRNA reduction. To validate our system, we orthogonally knocked down the fusogen MYMX (∼60% efficiency) (Figure S10), which significantly reduced myotube area as expected. These opposing phenotypes confirm that the hypertrophic effect of partial CREM knockdown is biologically robust. Functional knockdown of CREM in CPSCs selectively upregulated late‐stage differentiation drivers (*MYOG*, *MYHC*, *TMEM8C*, *MYMX*) [58] without affecting early commitment factors *MYOD1* and *MYF5*, and concomitantly reduced the expression of MSTN, FBXO32, and TRIM63, a well‐established negative regulator of muscle mass [59] and atrophy‐related E3 ubiquitin ligases [60], respectively, culminating in enhanced myotube formation. The coordinated downregulation of all three factors upon CREM depletion suggests that CREM functions as a transcriptional hub simultaneously maintaining multiple negative regulators of the myogenic program. Notably, in vivo knockdown of CREM resulted in a 29.96% increase in PMW and a 20.50% increase in PMP, substantially exceeding the approximately 11% increase in PMW reported following MSTN knockout in chickens [61]. This highlights CREM as a promising molecular target with considerable potential for genetic improvement of muscle mass in farm animals.

Collectively, this study demonstrates that postnatal myofiber hypertrophy is sustained by a coordinated interplay among myogenic subpopulations, FAPs, and immune cells within the muscle niche, and reveals that CREM acts as a transcriptional brake that restrains myofiber hypertrophy. Mechanistically, CREM directly binds and transcriptionally activates the *JUN* promoter, and the two factors cooperatively maintain the expression of key muscle growth inhibitors, including MSTN, FBXO32, and TRIM63, thereby limiting myofiber hypertrophy. However, whether CREM exerts similar regulatory roles in other vertebrate species remains to be directly demonstrated, warranting further cross‐species investigation. Future studies will therefore focus on validating the conservation of this transcriptional brake in mammalian muscle systems and elucidating the deeper mechanisms through which CREM coordinates its broader inhibitory network.

In conclusion, we successfully established a time‐resolved single‐nucleus transcriptomic resource for postnatal chicken muscle and functionally demonstrated that attenuation of CREM, a brake on myofiber hypertrophy, significantly promotes myofiber hypertrophy and muscle mass (20.50% increase in pectoralis major percent). Overall, these findings provide new insights into the transcriptional regulation of vertebrate skeletal muscle development and offer a valuable molecular target for strategies aimed at modulating the meat production performance in farm animals.

## 4 Experimental Methods

### 4.1 Ethics Statements

All experimental procedures involving the chickens in the present study were conducted in strict accordance with the guidelines established by the Ministry of Science and Technology (Beijing, China). Ethical approval for this research was granted by the Animal Welfare Committee of China Agricultural University (Approval No. AW71802202‐1‐2).

### 4.2 Experimental Birds and Pectoralis Major Sample Processing

The fertilized eggs of FG (WOD188 broilers) and SG (Rhode Island White layers) were gained from Beijing Huadu Yukou Poultry Breeding Co., Ltd. (Beijing, China) and were incubated with the temperature of 37.8°C and a relative humidity of 60% after disinfecting using 1% benzalkonium bromide solution. After hatching, all the birds were sexed, and the healthy males were raised in the Experimental Base of Poultry Genetic Resources and Breeding of China Agricultural University under the same conditions, provided with water and food ad libitum. Eight males of each experimental group were randomly selected at DOH, D7, D14, D21, D28, D35 and D42. The BW and PMW were carefully measured and recorded. The left side of pectoralis muscle tissue samples were quickly frozen in liquid nitrogen and stored at −80°C for total RNA extraction and snRNA‐seq. During tissue collection, visible surface fat was meticulously removed from the pectoralis major using fine forceps to minimize lipid interference. The right side was collected and fixed with 4% paraformaldehyde, embedded in paraffin, and subjected to H&E staining [32]. Histological characteristics of muscle fibers were quantified using MyoV software [62].

### 4.3 Nuclei Isolation and snRNA‐Seq on the 10× Genomics Platform

The frozen pectoralis major muscle samples of FG and SG at DOH, D14, D28, and D42 were homogenized in 2 mL of pre‐cooled Nuclei EZ Lysis buffer for 2 min at 4°C. The mixture was incubated on ice for 5 min with an additional 2 mL of lysis buffer. 2 mL of pre‐cooled 4% BSA was added to the mixture to terminate lysis, and the suspension was gently mixed. Samples were centrifuged at 300 ×g for 5 min at 4°C. The precipitate was resuspended in lysis buffer containing 4% BSA and incubated on ice for 3 min to reduce non‐specific binding. Cell debris was removed using debris removal solution (Miltenyi Biotec) according to the manufacturer's instructions. After a final centrifugation at 300 ×g for 5 min at 4°C, the nuclear pellet was resuspended in Nuclei Suspension Buffer (1× PBS, 0.07% BSA, and 0.1% RNase inhibitor). The suspension was filtered through a 20‐µm cell strainer (pluriSelect), and nuclei were counted with a Countess II automated cell counter (Invitrogen). The concentration of nuclei suspension was adjusted to 700–1200 nuclei/µL.

Single‐nuclei suspensions were loaded to 10× Chromium to capture approximately 8 000 nuclei per sample, following the manufacturer's instructions of 10× Chromium Single‐Cell 3’ Reagent Kit v3.1 (10× Genomics). Then, the cDNA synthesis, amplification, and library construction were performed. Libraries were quantified using a Qubit 4 Fluorometer (Thermo Fisher Scientific), and the fragment size distribution was validated with a Bioanalyzer 2100 (Agilent Technologies). All libraries were pooled and sequenced on an Illumina NovaSeq 6000 platform (LC‐Bio Technologies, Hangzhou, China) using paired‐end reads (150 bp × 2) in a multiplexed run, achieving a minimum sequencing depth of 20 000 reads per nucleus.

### 4.4 SnRNA‐Seq Data Filtering and Clustering

Raw sequencing data were converted to FASTQ format using Illumina bcl2fastq software (v5.0.1). Single‐cell gene expression matrices were generated with Cell Ranger (v7.2.0) [63] by aligning reads to the Ensembl GRCg7b chicken reference genome. Ambient RNA contamination was removed using CellBender (v0.3.0) [64]. Downstream analysis was performed using the Seurat package (v4.4.0). Low‐quality cells expressing fewer than 500 genes or exhibiting more than 25% mitochondrial transcripts were excluded. Putative doublets were identified and removed with DoubletFinder (v2.0.4) [65]. The remaining cells were normalized using NormalizeData. The FindVariableGenes function with the variance stabilizing transformation method in Seurat was used to identify highly variable genes cross nuclei. The top 2000 most variable genes were selected for downstream analysis to balance biological signal and computational efficiency. Dimensionality reduction was performed using PCA on the normalized expression matrix of these highly variable genes, followed by graph‐based clustering using FindNeighbors and FindClusters. Genes associated with the top 20 principal components were used for uniform manifold approximation and projection (UMAP) analysis and cluster identification with a resolution of 0.8.

### 4.5 Transcriptional Heterogeneity Analysis

To quantify and compare transcriptional heterogeneity of eight cell populations between the two chicken breeds across different time points, we computed a normalized heterogeneity score for each population using the variance‐to‐mean ratio (VMR) of gene expression levels per nucleus. Specifically, for each cell population, the VMR was calculated as the variance of log‐normalized expression values (scaled to 10 000 counts per nucleus) divided by their mean, excluding genes with zero or near‐zero expression (< 0.1 counts in > 95% of nuclei). This metric reflects the degree of transcriptional dispersion within a population: higher VMR indicates greater transcriptional variability. The resulting heterogeneity scores were compared between FG and SG samples for each cell population using the two‐sided Wilcoxon rank‐sum test, accounting for non‐normal distributions commonly observed in single‐nucleus data. All analyses were performed in R (v4.2.2).

### 4.6 Differential Gene Expression Analysis and Pathway Enrichment

To identify transcriptional differences between experimental groups within each cell subpopulation, differential gene expression analysis was performed using the Seurat's FindAllMarker function. Normalization and scaling were applied using default parameters. Differentially expressed genes were screened with the bimod statistical test, which is well‐suited for single‐cell data distributions. Genes expressed in at least 10% of cells in either the SG or FG group, with a |log_2_ fold change| > 0.26 and a *p*‐value < 0.01, were identified as differentially expressed genes. For downstream functional enrichment, differentially expressed gene lists were further filtered to prioritize biologically meaningful changes, including upregulation of pathway‐relevant regulators and transcription factors. GO biological process terms and KEGG pathways were analyzed using the clusterProfiler package (v4.6.1) [66], with enrichment significance threshold set at *p*‐value < 0.05.

### 4.7 Cell Trajectory Analysis

Pseudotime trajectories within the myogenic lineage cell populations were inferred using the Monocle 2 package (v2.22.0) [67]. The pseudotime trajectory was constructed using all differentially expressed genes (*q*‐value < 0.01) across myogenic lineage subpopulations. Dimensionality reduction was performed using the reduceDimension function with the DDRTree algorithm [68], and the orderCells () function was used for cell ordering. The root state was specified based on the biological context of the myogenic differentiation process. Specifically, the algorithm determined the overall cell ordering, and the root was assigned according to prior biological knowledge of the myogenic differentiation trajectory. Genes significantly associated with pseudotime were identified using the differentialGeneTest function. Moreover, BEAM was applied for branched‐dependent gene expression analysis [69].

### 4.8 Analysis of Co‐Expressed Gene Modules

Co‐expression network analysis was performed on the *MYBPC1* ^+^ myocytes, *KLHL40* ^+^ myocytes, and *AK1* ^+^ myocytes using the hdWGCNA package [70]. A gene set associated with stem cell differentiation (GO:0048863) was used for downstream analysis. After log transformation and normalization of gene expression values, genes detected in at least 10% of nuclei within the target subpopulation were retained. A gene co‐expression correlation matrix was then constructed, followed by weighted gene co‐expression network analysis and module detection. Module‐trait relationships were assessed to identify modules of interest, and hub genes within each module were determined based on eigenvector‐based intramodular connectivity (kME). Rather than applying an absolute kME threshold, genes within each module were ranked by their kME values, and the top 30 hub genes by connectivity rank were selected as representative key genes of the module using the GetHubGenes () function.

### 4.9 Intercellular Communication and Cell‐Cell Adhesion

Cell‐cell communication and ligand‐receptor analysis across the myogenic lineage, immune, and FAP subpopulations were performed using the CellChat package (v2.1.2) [71]. CellChat quantitatively inferred and analyzed intercellular communication networks from snRNA‐seq data using a curated database of known interactions among ligands, receptors, and their cofactors. Inference of cell type‐specific signaling communication was performed using mass action models. All significant interactions were visualized as chord diagrams and dot plots using CellChat, with edge thickness and dot size reflecting interaction strength and cluster abundance, respectively. The *P*‐values generated by the CellChat analysis assess the statistical significance of the existence of specific ligand‐receptor interactions, rather than testing for statistical differences in communication strength between the FG and SG.

### 4.10 Transcriptional Factors Analysis and Target Genes Prediction

Transcriptional factor analysis and CREM target gene prediction was performed based on the single‐nucleus expression matrix using the pySCENIC pipeline [72]. First, the pyscenic grn module was applied with the GRNBoost2 algorithm and a species‐specific transcription factor list to infer the transcription factor–target gene co‐expression network. Subsequently, the pyscenic ctx module was used in combination with the cisTarget database and motif‐transcription factor annotation files for regulon pruning. This step employed the –mask_dropouts parameter along with the default pySCENIC thresholds: rank_threshold = 5000, auc_threshold = 0.05, NES_threshold = 3.0, max_similarity_FDR = 0.001, and min_genes = 20. The genes retained in the pruned CREM regulon were defined as candidate CREM target genes.

### 4.11 RNA In Situ Hybridization

RNA in situ hybridization was performed on formalin‐fixed, paraffin‐embedded sections of pectoralis muscle from the two chicken breeds at D28 to visualize the spatial expression patterns of *TNNT3* and *CREM*. A validated amplification‐based FISH platform was employed, using target‐specific probes conjugated to distinct fluorophores, with hybridization and signal amplification carried out under standardized conditions to ensure reproducibility across samples. Tissue sections were processed through deparaffinization, antigen retrieval, permeabilization, and sequential hybridization steps, followed by counterstaining with DAPI and mounting with antifade reagent. Images were captured with a Pannormic MIDI II slide scanner from 3DHISTECH, and all experiments included no‐probe and scramble‐probe controls to confirm signal specificity. The targeted sequences of *TNNT3* and *CREM* used in this study are listed in Table S13.

### 4.12 CPSCs Isolation and Culture

CPSCs were isolated from the pectoralis major of 4‐day‐old broilers following humane procedures. Briefly, the pectoralis major tissues were dissected under sterile conditions, carefully removing adipose tissue, connective tissue, and blood vessels. Muscle fragments were minced and washed twice with PBS supplemented with 1% penicillin/streptomycin. Tissues were then digested into single cells using 0.25% trypsin (Gibco, USA) at 37°C for 5 min, and the reaction was halted by with the addition of complete DMEM/F12 medium (Gibco, USA) containing 10% fetal bovine serum (FBS, Gibco, USA) and 1% penicillin/streptomycin. The resulting cell suspension was passed through a 70 µm strainer followed by a 40 µm cell strainer to remove debris and large aggregates. Cells were resuspended in DMEM/F12 supplemented with 10% FBS and 1% penicillin/streptomycin, and subjected to two consecutive rounds of differential adhesion (3 h each) to deplete fibroblasts and other non‐myogenic cells. To induce myoblast fusion, the cell growth medium was switched to DMEM/F12 containing 2% horse serum (Gibco, USA) when the primary myoblasts were about 80% confluent.

### 4.13 Knockdown and Overexpression of *CREM* and *JUN*

The siRNAs targeting chicken *CREM* and *JUN* (Table S14) were synthesized by Sangon Biotech (Shanghai, China). For overexpression, the full‐length coding sequence of *CREM* and *JUN* was cloned into the expression vector of pcDNA3.1 (pcDNA3.1‐CREM and pcDNA3.1‐JUN, respectively), while pcDNA3.1‐Empty served as the empty vector control. The transfection of cells was conducted using the Lipofectamine 3000 Transfection Reagent (Invitrogen, Carlsbad, USA), following the manufacturer's protocol. Six hours post‐transfection, the medium was replaced with fresh growth medium, and cells were subsequently induced to differentiate.

### 4.14 RNA Extraction and Real‐Time Quantitative PCR

Total RNA was extracted from the pectoralis major and cells using TRIzol reagent (TaKaRa, Otsu, Japan), following standard procedures. Reverse transcription was performed using the PrimeScript RT Reagent Kit with gDNA Eraser (Takara, Kyoto, Japan). Real‐time quantitative PCR (RT‐qPCR) was carried out using TB Green Premix Ex Ta (Tli RNaseH Plus) kit (Takara, Kyoto, Japan) on the ABI7500 system. Each reaction was performed in technical triplicate. Relative gene expression levels were calculated using the 2^−ΔΔCt^ method [73], with the chicken *β‐actin* gene serving as the reference gene. The primers used in this study were designed using Premier 5 software, and the detailed information is listed in Table S15.

### 4.15 Western Blotting

Total tissue and cell protein were extracted using RIPA lysis buffer (Beyotime, Shanghai, China) and quantified by the BCA method (Beyotime, Shanghai, China). Equal amounts of protein were separated by SDS‐PAGE on 10% or 15% polyacrylamide gels and subsequently electrotransferred onto PVDF membranes (Millipore, Bedford, USA). After blocking with a dedicated blocking buffer (Beyotime, Shanghai, China) for 1 h at room temperature, membranes were probed with primary antibodies overnight at 4°C. Following three washes with TBST, membranes were incubated with HRP‐conjugated secondary antibodies (ZenBio, Chengdu, China) for 1 h at room temperature. Immunoreactive bands were detected by enhanced chemiluminescence (Beyotime, Shanghai, China) and imaged using a gel documentation system (Bio‐Rad). The complete list of antibodies used is available in Table S16.

### 4.16 Immunofluorescence

CPSCs at DM3 were seeded into 24‐well plates and fixed in 4% paraformaldehyde for 20 min, permeabilized with 0.1% Triton X‐100 for 10 min, and blocked in goat serum for 1 h. Cells were incubated with an anti‐MyHC antibody (Santa Cruz, CA, USA; 1:500) overnight at 4°C, followed by 2 h incubation with a diluted fluorescent secondary antibody (ZenBio, Chengdu, China; 1:1000) at 37°C in the dark. Subsequently, washed cells were mounted with DAPI‐contained anti‐fade medium (Beyotime, Shanghai, China). Immunofluorescence images were captured using a fluorescence microscope. The relative myotube diameter and area was calculated as the percentage of MyHC fluorescence area to the DAPI fluorescence area. These calculations are performed by the Image pro plus software (v6.0).

Paraffin‐embedded pectoralis muscle sections were deparaffinized and subjected to antigen retrieval. After blocking with 10% goat serum for 30 min, sections were incubated overnight at 4°C with a primary antibody cocktail containing anti‐KUL01 (Thermo Fisher Scientific, Waltham, USA; 1:100). Following primary incubation, sections were incubated at room temperature in the dark for 50 mins with a secondary antibody. Sections were then added with WGA (Sigma, Aldrich, USA; 1:200) at 37°C in the dark for 30 min to label cell membranes. Nuclei were counterstained with DAPI, and images were acquired using a fluorescence microscope.

### 4.17 Dual‐Luciferase Reporter Assay

To analyze *JUN* promoter activity and validate direct *CREM* occupancy, the *JUN* promoter sequence containing either the wild‐type or mutant CREM‐binding site was synthesized and subcloned into the pEZX‐FR01 dual‐luciferase reporter vector (GenePharma, Shanghai, China). CPSCs were seeded in 48‐well plates and transfected with the wild‐type or mutant reporter constructs alone or co‐transfected with the pcDNA3.1‐CREM overexpression vector, respectively. Firefly and Renilla luciferase activities were measured sequentially using the Dual‐Luciferase Reporter Gene Assay Kit (Beyotime, Shanghai, China) after 48 h of transfection, and the relative luciferase activity was calculated as the ratio of firefly to Renilla luminescence, with Renilla luciferase serving as an internal control to normalize for transfection efficiency.

### 4.18 Chromatin Immunoprecipitation Assay

To investigate the direct binding of *CREM* to the *JUN* promoter region, a FLAG‐tagged CREM expression plasmid (Flag‐CREM, pcDNA3.1) was constructed and transfected into CPSCs. Chromatin immunoprecipitation (ChIP) assays were performed using a commercial kit (BersinBio, Guangzhou, China) according to the manufacturer's instructions. Briefly, cells were cross‐linked with 1% formaldehyde for 10 min at room temperature, and the reaction was quenched by adding 1.375 mol/L glycine. Cells were subsequently lysed, and chromatin was sonicated into fragments ranging from 200 to 500 bp. Immunoprecipitation was carried out overnight at 4°C using an anti‐FLAG antibody (Zenbio, Chengdu, China; 1:500), while normal IgG (Zenbio) served as a negative control. After reversal of cross‐links and proteinase K digestion, the immunoprecipitated DNA was purified and subjected to PCR amplification using primers targeting the predicted CREM‐binding site within the *JUN* promoter. PCR products were separated by agarose gel electrophoresis and visualized using a Bio‐Rad Gel Doc XR+ Imaging System.

### 4.19 Lentivirus Production and Transduction

The siRNA targeting *CREM* was cloned into the pLV3ltr‐Puro‐U6 vector (LV‐si‐CREM), with an empty vector serving as the negative control (LV‐si‐NC). The resulting constructs were co‐transfected with the psPAX2 and pMD2.G packaging vectors into HEK293T cells to generate lentiviral particles. Following purification, the lentiviral titer was adjusted to 5 × 10^8^ Transduction Units (TU)/mL. A total volume of 100 µL, containing 1 × 10^7^ TU lentivirus, 0.1 µL of 4 mg/mL Polybrene (Tsingke Biotechnology, Beijing, China), and an appropriate volume of PBS, was directly injected into the pectoralis major of 7‐day‐old male small white‐feather broilers from a developing line (*n* = 6). This line exhibits a moderate growth rate compared with fast‐growing broilers (WOD188) and was specifically selected for the in vivo experiment to provide greater sensitivity for detecting the promotive effects on myofiber hypertrophy after *CREM* knockdown. A booster injection was administered at D14 using the same dose. Birds were maintained until D21, at which point pectoralis major samples were harvested for subsequent analyses including qRT‐PCR, western blotting, H&E staining, and myofiber CSA quantification. Six males per group were used for the in vivo phenotypic assessments following lentiviral injection, and ten microscopic fields per individual for the CSA measurements.

### 4.20 Statistical Analysis

All statistical analyses and visualization were performed in R (v.4.5.1) and GraphPad Prism (v10.0). Data are represented as mean ± SEM. The sample size (n) for each analysis represents the number of independent biological replicates (*n* = 8 per group for phenotypic determination, *n* = 6 per group for in vivo experiments, and *n* = 3 per group for in vitro assays). Comparisons between two groups were assessed using a two‐tailed unpaired Student's *t* test, and comparisons among three groups were assessed using one‐way analysis of variance followed by Tukey's honest significant difference post hoc test. Transcriptional heterogeneity scores between FG and SG groups were compared using a two‐sided Wilcoxon rank‐sum test. Statistical significance was defined as follows: ^*^ *p* < 0.05; ^**^ *p* < 0.01; different capital/lowercase letters indicate *p* < 0.01/*p* < 0.05, respectively. Details on the statistics are indicated in the figure legends.

## Author Contributions

**N.Y. and H.D.Y**. conceived and designed the project. **N.Y**. supervised the project. **S.G**. conducted the sample collection, data analyses, experiment implementation, and figure visualization. **X.Y.Z**. performed cell experiment and data analyses. **Z.Y.J**. assisted in experimental verification. **C.L.W., C.Y.W., Q.L.W., Q.H., F.R.L., and C.J.S**. helped collect samples. **C.L.W. and S.H.W**. assisted in data analyses. **J.Y.L**. carried out the rearing of chickens. **S.G. and X.Y.Z**. wrote the initial manuscript. All authors read and approved the final manuscript.

## Funding

This work was funded by the National Key Research and Development Program of China (2022YFF1000200) and the 2115 Talent Development Program of China Agricultural University (00109015).

## Conflicts of Interest

The authors declare no conflicts of interest.

## Supporting information




**Supporting File 1**: advs78534‐sup‐0001‐SuppMat.docx.

**Supporting File 2**: advs78534‐sup‐0002‐TableS1‐S16.xlsx.

## Data Availability

The data that support the findings of this study are openly available in China National GeneBank at https://db.cngb.org/mycngbdb/submissions/project, reference number CNP0009544.
