# Supplementary material for: Single‐Nucleus RNA Sequencing Identifies CREM as a Brake on Myofiber Hypertrophy in Chickens
Source: Adv Sci (Weinh). 2026 Oct 28:e78534. Online ahead of print. doi: 10.1002/advs.78534 (PMC13715926; doi:10.1002/advs.78534)
Supplement: Supplementary file 1 [file ADVS-9999-e78534-s002.docx]

Supporting Information

**Title:**

**Single-Nucleus RNA Sequencing Identifies CREM as a Brake on Myofiber Hypertrophy in Chickens**

**Authors:**

*Shuang Gu*, *Xiyu Zhao*, *Zheyi Jiang*, *Chaoliang Wen*, *Chaoyi Wang*, *Qiulian Wang*, *Qiang Huang*, *Fangren Lan*, *Shenghui Wu*, *Junying Li*, *Congjiao Sun*, *Huadong Yin**, *Ning Yang**

**Figure S1. Phenotypic characterization of muscle growth and myogenic marker expression in FG and SG. (A)** Body weight (BW) of fast-growing broilers (FG) and slow-growing layers (SG) at day of hatch (DOH), day 7 (D7), D14, D21, D28, D35, and D42. Data are presented as mean ± SEM (n = 8). Statistical significance was determined by two-tailed unpaired Student’s t-test. **(B)** Dynamic changes in myofiber density (MFD) from DOH to D42 in FG and SG. Data are represented as mean ± SEM (n = 8). Statistical significance was determined by two-tailed unpaired Student’s t-test. **(C)** Total number of myofibers (TNM) in the pectoralis major muscle of FG and SG. Red dots indicate the group mean (n = 8). Statistical significance was determined by two-tailed unpaired Student’s t-test. **(D, E)** mRNA expression levels of *MYOD1* (D) and *PAX7* (E) in pectoralis major at DOH, D14, D28, and D42, measured by Real-time quantitative PCR (RT-qPCR). Data are normalized to *β-actin* and red dots indicate the group mean (n = 6). Statistical significance was determined by two-tailed unpaired Student’s t-test**. (F)** Transcriptional heterogeneity of each population between FG and SG at DOH, D14, and D28. The sample size for each group represents the number of nuclei within the respective cell population. Transcriptional heterogeneity scores between FG and SG groups were compared using a two-sided Wilcoxon rank-sum test. ^*^*P* < 0.05; ^**^*P* < 0.01; n.s.= not significant.

**Figure S2. Differential gene expression and pseudotime trajectory analysis of myogenic subpopulations. (A)** Differential gene expression between FG and SG within each myogenic subpopulation, computed using the MAST statistical framework. **(B)** Pseudotime distribution of myogenic subpopulations across individuals, illustrating consistency of trajectory inference. **(C)** Branch expression analysis modeling (BEAM) applied to branch point 2 to identify genes associated with divergent fate decisions.

**Figure S3. Transcriptional heterogeneity and functional divergence of FAP subpopulations during postnatal muscle development. (A)** Expression of top signature genes defining the seven subpopulations of fibro-adipogenic progenitors (FAPs). **(B)** Temporal changes in the proportion of each FAP subpopulation in FG and SG across four developmental timepoints (DOH, D14, D28, D42). **(C)** Number of differentially expressed genes (DEGs) between FG and SG within each FAP subpopulation at DOH, D14, D28, and D42. **(D)** GO enrichment analysis of differentially expressed genes in matrix FAPs at D14. **(E, F)** GO (E) and KEGG (F) pathway enrichment analysis of differentially expressed genes in matrix FAPs at D28.

**Figure S4. Dynamic remodeling of immune cell subpopulations and breed-associated transcriptional divergence.**

**(A)** Temporal dynamics of immune cell subpopulation proportions in FG and SG from DOH to D42. **(B)** Distribution of the four immune cell subpopulations in FG and SG chickens at D14 and D28. **(C)** Differential gene expression between FG and SG within each subpopulation. Red dots above the axis indicate genes upregulated in FG; red dots below indicate genes upregulated in SG. **(D)** GO enrichment analysis of differentially expressed genes in macrophages and fetal macrophages from FG and SG. **(E)** KEGG pathway enrichment analysis of differentially expressed genes in macrophages and fetal macrophages from FG and SG.

**Figure S5. Intercellular communication networks among myogenic, FAP, and immune cell subpopulations. (A)** Total number and strength of inferred cell-cell interactions among myogenic subpopulations, FAP subpopulations, fetal macrophages, and macrophages in FG and SG across four developmental timepoints. **(B)** Network map of interaction counts between specific subpopulations. Red edges indicate more interactions in FG than SG; blue edges indicate more in SG than FG. **(C, D)** Ligand-receptor interactions emanating from fetal macrophages in FG (C) and SG (D). Arrows point from ligand expressing to receptor expressing subpopulations. **(E, F)** Ligand-receptor interactions emanating from macrophages in FG (E) and SG (F). **(G)** IGF signaling network across cell subpopulations, highlighting active ligand-receptor pairs. **(H, I)** Interactions received by *AK1*^+^ myocytes in SG (H) and FG (I).

**Figure S6.** Spatial expression of LAMA2, LAMA4, and TENM4 expression across myogenic subpopulations.

**Figure S7. Identification of key regulatory modules and transcription factors governing myogenic progression. (A)** Selection of the optimal soft threshold for hierarchical deterministic weighted gene co expression network analysis (hdWGCNA). **(B)** Dendrogram showing clustered co-expression modules generated by hdWGCNA across myogenic subpopulations. **(C)** Dot plot of module eigengene expression across specific subpopulations. Modules most highly expressed in *AK1*^+^ myocytes is highlighted with hub gene networks. **(D)** UMAP visualization of the three candidate transcription factors (CREM, JUN, and JUND). **(E)** Expression levels of *CREM*, *JUN*, and *JUND* in myogenic subpopulations of FG and SG across four developmental timepoints (DOH, D14, D28, D42). **(F)** The difference in the proportion of CREM-positive nuclei relative to total nuclei between FG and SG. Data are presented as mean ± SEM. The sample size represents n = 6 independent fields of view per group. Statistical significance was determined by a two-tailed unpaired Student’s t-test. n.s.= not significant. **(G)** Venn diagram showing the intersection of genes within the red module identified by hdWGCNA and predicted downstream target genes of CREM.

**Figure S8. Expression dynamics of myogenic regulatory factors and siRNA validation in CPSCs. (A–C)** mRNA expression of *MYOD1* and *MYF5* (A), *MYOG* and *MYHC* (B), and *TMEM8C* and *MYMX* (C) across GM, DM1, DM2, and DM3 stages in chicken primary satellite cells (CPSCs). Data are presented as mean ± SEM (n = 3). **(D)** Knockdown efficiency of three independent si-CREM constructs in CPSCs. Red dots indicate the group mean (n = 3). **(E-F)** mRNA expression of *MYOD1* (E) and *MYF5* (F) in si-NC and si-CREM groups. Red dots indicate the group mean (n = 3). **(G)** Protein expression levels of MyoG in si-NC and si-CREM groups. Data are presented as mean ± SEM (n = 3). **(H)** Quantification of relative myotube area and diameter in si-NC and si-CREM groups. Data are presented as mean ± SEM (n = 3). Comparisons between two groups were assessed using a two-tailed unpaired Student’s t test, and comparisons among four groups were assessed using one-way analysis of variance followed by Tukey’s honest significant difference post hoc test. ^*^*P* < 0.05; ^**^*P* < 0.01; n.s.= not significant; different lowercase and uppercase letters indicate significant (*P* < 0.05) and highly significant (*P* < 0.01) differences, respectively; identical letters indicate no significant difference (*P* > 0.05).

**Figure S9. Transcriptional brake function validation of CREM through rescue experiments and in vivo lentiviral silencing. (A)** Site-specific PCR amplification confirming the authenticity of the CREM-binding site within the *JUN* promoter. Schematic illustration of wild-type and mutant *JUN* promoter dual-luciferase reporter constructs. **(B)** Knockdown efficiency of three independent si-JUN constructs in CPSCs. Red dots indicate the group mean (n = 3). **(C)** Quantification of relative myotube diameter across the si-NC + pcDNA3.1-Empty, si-JUN + pcDNA3.1-Empty, and si-JUN + pcDNA3.1-CREM groups. Data are presented as mean ± SEM (n = 3). **(D)** Western blot images of CREM, c-Jun, P-c-Jun, MyHC, MyoG, MSTN, FBXO32, and TRIM63 in si-NC + pcDNA3.1-empty, si-NC + pcDNA3.1-JUN, and si-CREM + pcDNA3.1-JUN groups. **(E)** Quantification of protein expression levels across the three groups. Data are presented as mean ± SEM (n = 3). **(F)** Quantification of relative myotube diameter across the si-NC + pcDNA3.1-Empty, si-NC + pcDNA3.1-JUN, and si-CREM + pcDNA3.1-JUN groups. Data are presented as mean ± SEM (n = 3). **(G)** Body weight (BW) of LV-si-NC and LV-si-CREM birds at D7 and D21. Red dots indicate the group mean (n = 6). **(H)** mRNA expression levels of JUN in LV-si-NC and LV-si-CREM groups. Red dots indicate the group mean (n = 3). **(I)** Quantification of protein expression levels of MyHC and MyoG in LV-si-NC and LV-si-CREM birds. Data are presented as mean ± SEM (n = 3). Comparisons between two groups were assessed using a two-tailed unpaired Student’s t test, and comparisons among three groups were assessed using one-way analysis of variance followed by Tukey’s honest significant difference post hoc test. ^*^*P* < 0.05; ^**^*P* < 0.01; n.s.= not significant.

**Figure S10. Orthogonal validation using MYMX. (A)** Knockdown efficiency of three MYMX-specific siRNAs in chicken primary satellite cells. Data are presented as mean ± SEM (n = 3). **(B)** Representative images of myotube differentiation in CPSCs transfected with the indicated siRNAs. Scale bar, 100 μm. **(C)** Quantification of relative myotube area in CPSCs transfected with the indicated siRNAs. Data are presented as mean ± SEM (n = 3). Comparisons among four groups were assessed using one-way analysis of variance followed by Tukey’s honest significant difference post hoc test. Different capital letters indicate highly significant differences (*P* < 0.01).

**Table S1.** Quality control metrics for snRNA-seq across all samples.

**Table S2.** Top 50 signature genes of each subpopulation of myogenesis lineage cells.

**Table S3.** Differentially expressed genes between FG and SG across myogenic subpopulations.

**Table S4.** Differentially expressed genes between FG and SG across fibro-adipogenic progenitor subpopulations.

**Table S5.** Differentially expressed genes between FG and SG across macrophage subpopulations.

**Table S6.** Gene set related to stem cell differentiation (GO:0048863).

**Table S7.** Genes of the seven modules.

**Table S8.** Correlations between modules and stemness.

**Table S9.** Three gene sets were integrated to identify transcriptional factors.

**Table S10.** Correlations between transcription factors and red module.

**Table S11.** Overlap of target genes and genes in red module.

**Table S12.** Top 10 enriched KEGG pathways for the 63 candidate genes.

**Table S13** Probe sequence information for RNA in situ hybridization.

**Table S14.** Sequences used for siRNA and plasmid-based overexpression.

**Table S15.** The primers used in RT-qPCR analysis.

**Table S16.** Information of antibodies.
